# Mechanism of Secondary Ganglioside and Lipid Accumulation in Lysosomal Disease

**DOI:** 10.3390/ijms21072566

**Published:** 2020-04-07

**Authors:** Bernadette Breiden, Konrad Sandhoff

**Affiliations:** Membrane Biology and Lipid Biochemistry Unit, LIMES Institute, University of Bonn, 53121 Bonn, Germany; bbreiden@gmx.de

**Keywords:** Niemann–Pick disease, mucopolysaccharidosis, cascade model, inhibitors of ganglioside catabolism, cholesterol as inhibitor, sphingomyelin as inhibitor, gangliosidoses

## Abstract

Gangliosidoses are caused by monogenic defects of a specific hydrolase or an ancillary sphingolipid activator protein essential for a specific step in the catabolism of gangliosides. Such defects in lysosomal function cause a primary accumulation of multiple undegradable gangliosides and glycosphingolipids. In reality, however, predominantly small gangliosides also accumulate in many lysosomal diseases as secondary storage material without any known defect in their catabolic pathway. In recent reconstitution experiments, we identified primary storage materials like sphingomyelin, cholesterol, lysosphingolipids, and chondroitin sulfate as strong inhibitors of sphingolipid activator proteins (like GM2 activator protein, saposin A and B), essential for the catabolism of many gangliosides and glycosphingolipids, as well as inhibitors of specific catabolic steps in lysosomal ganglioside catabolism and cholesterol turnover. In particular, they trigger a secondary accumulation of ganglioside GM2, glucosylceramide and cholesterol in Niemann–Pick disease type A and B, and of GM2 and glucosylceramide in Niemann–Pick disease type C. Chondroitin sulfate effectively inhibits GM2 catabolism in mucopolysaccharidoses like Hurler, Hunter, Sanfilippo, and Sly syndrome and causes a secondary neuronal ganglioside GM2 accumulation, triggering neurodegeneration. Secondary ganglioside and lipid accumulation is furthermore known in many more lysosomal storage diseases, so far without known molecular basis.

## 1. Introduction

Many classical lipidoses, previously known as thesaurismosis [1,2], are caused by monogenic defects in lysosomal sphingolipid catabolism. The inherited defect of a single, usually promiscuous lysosomal hydrolase blocks sphingolipid catabolism, and triggers a progressive lysosomal accumulation of its non-degradable substrates. Besides these primary storage compounds, the analysis of patients’ tissues revealed, however, the additional accumulation of secondary material without any defect in its catabolic pathway, greatly modifying cellular metabolic pathways and inducing additional pathology in patients. Well known is the secondary accumulation of gangliosides (GGs) and glycosphingolipids (GSLs) in Niemann–Pick disease types A, B, and C [3,4] and in mucopolysaccharidoses (MPSs) like Hurler, Hunter, Sanfilippo, and Sly syndrome [5]. In the case of a single inherited hydrolase defect, the primary accumulation of its known substrates is accompanied by a secondary storage apparently unrelated to the genetic defect. This secondary storage may arise from reasons and mechanisms yet unknown, but may be itself critically involved in the pathogenesis of the disease. 

This review summarizes recently identified molecular and membrane associated topological mechanisms that trigger cascades of metabolic errors within the lysosomal compartment generating an additional, secondary accumulation of gangliosides and other lipids. In reconstitution experiments, we identified stimulators and inhibitors that strongly affect various catabolic sphingolipid pathways in the lysosome. Primary lysosomal storage compounds like sphingomyelin, cholesterol, and chondroitin sulfate were identified as inhibitors of GSL-catabolism triggering a secondary GSL-accumulation in Niemann–Pick diseases and in MPS [6,7,8,9]. For instance, sphingomyelin is an effective inhibitor of cholesterol secretion from the late endosomal and lysosomal compartment explaining the enormous secondary lysosomal cholesterol accumulation in Niemann–Pick disease types A and B [10]. Secondary GSL accumulation, however, is also known in many other lysosomal storage diseases (LSDs), so far, mostly with unknown mechanistic basis. Causes and reasons of secondary GSL accumulation and its pathological consequences are discussed below.

## 2. Primary Storage Compounds in Gangliosidoses and Historical Aspects

The clinical picture of amaurotic idiocy, a fatal inherited form of progressive mental deficiency associated with early blindness, was known for a long time [11,12]. Pathological, histochemical, and chemical investigations eventually identified the accumulation of novel glycolipids in the brain tissue of infantile patients, named “Gangliosides” by Ernst Klenk [13]. Chemical, metabolic, enzymatic, and genetic investigations clarified the molecular, genetic, and cellular basis of these LSDs. Currently, early therapeutic approaches are under way for these inherited neurodegenerative diseases, especially a gene replacement approach for infantile patients. Two types of GG storage disorders are known (Table 1). GM1 gangliosidosis, caused by an inherited deficiency of the GM1 degrading lysosomal hydrolase acid β-galactosidase (EC 3.2.1.23), and several forms of GM2 gangliosidosis that result from defects in the GG GM2 catabolizing β-hexosaminidases and the hexosaminidases A (Hex A) associated GM2 activator protein (GM2AP), a lipid binding and transfer protein [8,14].

In 1881, Warren Tay [15] described an infantile patient with the clinical diagnosis of amaurotic idiocy, suffering from blindness and loss of its cognitive capabilities, now called Tay–Sachs disease (TSD). Its major neuronal storage compound, the GM2 (Table 1), is a degradation product of GG GM1 [16,17], the structure of which was elucidated in 1963 [18]. The elucidation of the enzyme-catalyzed GM2 hydrolysis was complex and tedious [19]. A Hex A-deficiency observed in the postmortem brain tissue of a single TSD patient in 1967 could not have been published. It was questioned by the analysis of another infantile “TSD patient” with GM2 storage in the brain that showed no enzyme deficiency, but an elevation of both, Hex A and Hex B activity levels in its postmortem brain tissue [20,21]. The latter patient was identified later as the first one to suffer from the defect of an essential lipid-binding cofactor of GG GM2 hydrolysis, the GM2AP, and not from an enzyme deficiency [22], see below. 

After receiving postmortem material of two additional patients, we confirmed the Hex A deficiency in TSD [20], which was also demonstrated by [23]. In vitro experiments proved the basic assumption, that Hex A indeed splits GM2 slowly in the presence of an appropriate anionic detergent to release its terminal sugar to form the minor GG GM3 [24].

Two types of catabolic GG disorders are known (Table 1). GM1 gangliosidosis is caused by a deficiency of the GM1 degrading lysosomal hydrolases acid β-galactosidase (EC 3.2.1.23). GM2 gangliosidoses are a group of disorders that result from defects in digestion of GG GM2 and related glycolipids by β-hexosaminidases (EC 3.2.1.52) and the GM2AP [8,25]. Both gangliosidoses are progressive neurodegenerative diseases previously diagnosed as “amaurotic idiocy” [16,26].

### 2.1. GM2 Gangliosidoses

GM2 gangliosidoses comprise four inherited neurodegenerative disorders (TSD, B1 variant of GM2 gangliosidosis, Sandhoff disease (SD), and GM2AP deficiency (AB variant)). All of these disorders are based on defects in the degradation of GM2 and related glycolipids by the β-hexosaminidases (EC 3.2.1.52) and the GM2AP [21,26]. The β-hexosaminidases are a combination of two subunits (α or β). Hex A consisting of an α- and a β-subunit cleaves terminal β-glycosidically linked *N*-acetylglucosamine and *N*-acetylgalactosamine residues from negatively charged and uncharged glycoconjugates. Hex B contains two β-subunits (ββ) and Hex S two α-subunits (αα) (Table 1). Hex B splits uncharged substrates such as the GSLs GA2, globoside (Gb_4_Cer), and oligosaccharides with terminal *N*-acetylhexosamine residues. Hex S is involved in the degradation of glycosaminoglycans, sulfated glycolipid SM2a, and glycolipid GA2 and GM2 [27].

#### 2.1.1. Tay–Sachs Disease (B Variant) and B1 Variant

TSD (B variant) is caused by α-chain mutations resulting in a deficiency of Hex A and Hex S activities, but keeps a normal Hex B activity [21]. The main neuronal storage compounds are the GG GM2 and its sialic acid free residue, the GSL GA2, minor accumulating metabolites are lyso-GM2 and SM2a [28].

Some patients generate a partially defective α-chain, which binds the β-subunit to generate a defective αβ-dimer (a defective Hex A), which is, however, still active against neutral substrates but has lost its catabolic activity against GM2 [33,34]. This variant is also called B1 variant of GM2-gangliosidosis. In contrast to TSD, Hex A of variant B1 patients is still active with neutral substrates like the glycosphingolipid GA2 and synthetic MUF (4-methylumbelliferone)-substrate, MUF-β-GlcNAc, but has lost its activity against anionic substrates like the main neuronal storage lipid, ganglioside GM2, and the soluble fluorogenic 4-methylumbelliferyl-6-sulfo-2-acetamido-2-deoxy-β-d-glycopyranoside (MUGS) substrate. Therefore, it differs from infantile TSD that lost Hex A activities against both, neutral and anionic substrates.

In contrast to the human clinical phenotype, mouse models for TSD differ severely in their phenotypes. The mouse model lacking Hex A and Hex S, shows no significant neurological phenotype. Mouse sialidase has some low activity, more than human enzyme, to accept GM2 as substrate, converting it slowly to GA2 [35], which is further degraded by the still active Hex B in the TSD mice.

In vitro studies have shown that sulfatide SM2a is also degraded by Hex A and Hex S in the presence of GM2AP [27]. Their functional deficiency triggers an accumulation of SM2a in TSD liver as well as in kidneys of the TSD mouse model [27].

#### 2.1.2. Sandhoff Disease (SD) (0 Variant)

Another genetic variant of amaurotic idiocy with a clinical picture typical of TSD, but exposing additional visceral involvement and an additional lipid storage of globoside in the visceral organs was discovered in 1967 [29,30] and described as Variant 0 of GM2 gangliosidosis [21], now called “Sandhoff disease” (SD). It is characterized by an inherited deficiency of both major hexosaminidases, Hex A and Hex B, triggered by a genetic defect of their common β-subunit.

In SD, the functional loss of both, Hex A and Hex B, causes an increased storage of uncharged glycolipid GA2 in the brain besides GG GM2 and an additional accumulation of globoside (globotetraosylceramide) and oligosaccarides in visceral organs [21,26]. The clinical phenotype is similar to TSD, but with additional mucopolysacchariduria in visceral organs and secretion of oligosaccharides into the urine.

In accordance with infantile patients, the SD mouse, lacking Hex A and Hex B, shows a severe neurological phenotype [36,37].

#### 2.1.3. GM2 Activator Protein Deficiency (AB Variant)

**Figure 1:**
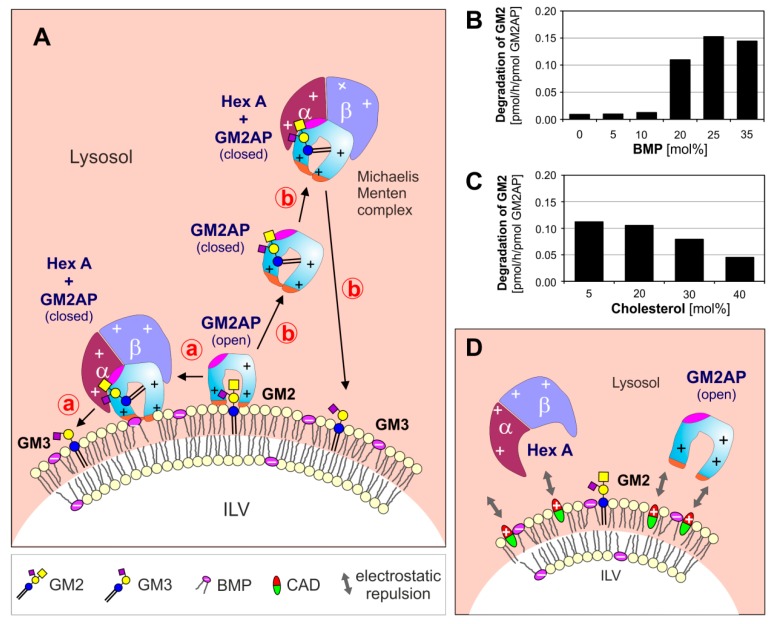
(**A**) Model for enzymatic digestion of membrane bound GM2 by Hex A, assisted by GM2AP at the surface of ILV at low, lysosomal pH-values. The open and empty GM2AP conformation binds to the membrane, e.g., by affinity to its lipid ligand and charge dependent interaction of the cationic protein (+, positively charged) with negatively charged membrane lipids (−, negatively charged) like BMP. Thereafter, the activator can interact with the ceramide portion of the GM2-ligand, which can move inside the hydrophobic cavity of the GM2AP, exposing the glycan chain of the GM2 to the water-soluble Hex A for hydrolysis. At this point, the conformation of the lipid-loaded activator may change to the closed one, thus the complex becomes more water soluble and can either stay at the surface of the membrane (pathway **ⓐ**) or leave the membrane (pathway **ⓑ**). (**B**, **C**) The GM2 hydrolysis is affected by membrane lipids: (**B**) anionic lipids e.g., BMP stimulate and (**C**) cholesterol inhibits GM2 degradation [38]. (**D**) CADs reaching the lysosome behave like cationic amphiphilic lipids, insert into the membrane surface of the intralysosomal luminal vesicles (ILVs) and start to compensate their negative surface charge. This results in a decreasing electrostatic attraction between proteins and ILVs, and an increasing repulsion between positively charged lysosomal proteins and the CAD-containing ILV-membrane. BMP: bis(monoacylglycero)phosphate, CADs: cationic amphiphilic drugs, Chol: cholesterol, GM2AP: GM2 activator protein, Hex A: β-hexosaminidase A, ILV: intralysosomal luminal vesicles.

An ultra-rare neurodegenerative genetic disease with GM2 as the main neuronal storage compound is caused by an inherited deficiency of an essential lipid binding and transfer glycoprotein, the GM2AP, having, however, about normal levels of functional Hex A, Hex B, and Hex S activities [22]. The concept, that both are needed, a soluble hydrolase and a corresponding lipid binding or sphingolipid activator protein (SAP), was found to be correct for the physiological degradation of many sphingolipids (Figure 1) [8].

### 2.2. GM1 Gangliosidosis

GM1 gangliosidosis was discovered by identifying GG GM1 and GSL GA1 as the main storage compounds in the postmortem brain tissue of a patient with infantile amaurotic idiocy [16,17]. The proposed block, a functionally deficient GM1-β-galactosidase in the catabolic pathway of GM1 was proven later by O’Brien et al. [26,39]. As many other lysosomal hydrolases, the GM1-β-galactosidase is a promiscuous glycosidase, which cleaves many oligosaccharides. Its deficiency triggers the accumulation of many other glycan substrates in GM1 gangliosidosis [40].

GM1 gangliosidosis is a progressive neurodegenerative disease due to absence or defective function of lysosomal acid β-galactosidase (E.C. 3.2.1.23), resulting in a storage of GM1 and its sialic acid-free derivate GA1. β-Galactosidase is part of a lysosomal multienzyme complex, containing also a sialidase and cathepsin A, the so-called protective protein. Another disease caused by mutations in the β-galactosidase gene *GBL1* is mucopolysaccharidosis type IVB called Morquio type B disease. These mutations are different from those of GM1 gangliosidosis, and lead to a changed substrate specificity of the enzyme, thereby resulting in major accumulation of galactose containing keratan sulfate and oligosaccharides [5,25].

In vitro studies have shown that two SAPs, GM2AP and saposin (Sap) B, redundantly stimulate the GM1 hydrolysis by β-galactosidase [41]. Therefore, neither a defect of GM2AP nor of Sap B causes a GM1 accumulation, since the one remaining efficiently facilitates the reaction.

## 3. Lysosomal Catabolism of GGs

**Figure 2:**
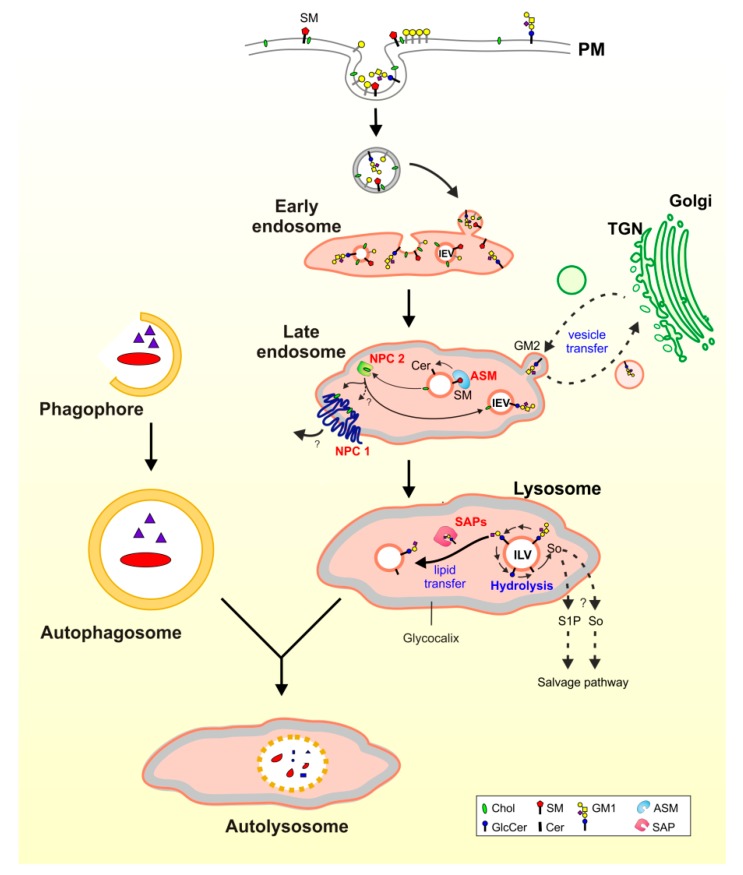
Lipids from the plasma membrane (PM) are degraded after endocytosis and internalization into intraendosomal luminal vesicles (IEVs) and intralysosomal luminal vesicles (ILVs). In the lysosome, ILV-bound (glyco-)sphingolipids are catabolized in a stepwise manner. Functional defects of any catabolic step cause an accumulation of the undegradable substrates in the lysosomes. The increasing lysosomal storage can trigger a reduced ability of lysosomes to fuse with autophagosomes, attenuating autophagy. ASM: acid sphingomyelinase, Cer: ceramide, Chol: cholesterol, GlcCer: glycosylceramide, IEV: intraendosomal luminal vesicle, ILV: intralysosomal luminal vesicle, NPC: Niemann–Pick disease type C protein, PM: plasma membrane, S1P: spingosine-1-phosphate, SAP: sphingolipid activator protein, SM: sphingomyelin, So: sphingosine.

Surfaces of mammalian neurons are enriched in GGs of the a- and b-ganglio-series (e.g., GM1a, GD1a, GD1b, GT1a, GT1b, and polysialo-GGs), carrying up to six sialic acid residues [9]. Desialylation of complex polysialo-GGs to eventually generate GG GM1 is catabolized mainly by three membrane-bound sialidases with overlapping substrate specificities and differing subcellular location, neuraminidase NEU1, NEU3, and NEU4 [42,43,44]. The plasma membrane-bound NEU3 is the key enzyme for degradation of polysialo-GGs and is involved in many surface phenomena, whereas NEU1 is the major sialidase of endosomes and lysosomes to hydrolyze polysialo-GGs to generate GM1 [45]. Catabolism of GM1 proceeds at the surface of intralysosomal luminal vesicles (ILVs) (see below) in a stepwise manner and is catalyzed by soluble lysosomal glycoproteins, which are hydrolases and lipid-binding proteins, the SAPs [8] (Figure 2).

In the lysosomes, NEU1 is part of a multienzyme complex together with the protective protein/cathepsin A, a stabilizing protein for NEU1, and the GM1 hydrolyzing β-galactosidase [25]. Its inherited defects in GM1-gangliosidosis cause mainly a progressive accumulation of GG GM1 and its sialic acid free residue GA1 in the nervous system [8,14], triggering a neurodegenerative disease. In contrast to most membrane-bound sialidases, the soluble GM1-β-galactosidase needs an essential cofactor, a lipid-binding and transfer protein, either GM2AP or Sap B [25,41] to remove the terminal galactose residue from membrane-bound GM1 to generate membrane-bound GM2. Genetic defects of NEU1 trigger a sialidosis and impair the lysosomal catabolism of sialylated metabolites causing their accumulation [46], whereas inherited deficiencies of the stabilizing protein lead to a progressive accumulation of GM1, other glycolipids and oligosaccharides [47,48].

In human tissues, GM2 degradation proceeds mainly with the removal of the terminal *N*-acetylgalactosamine residue by Hex A with the help of the lipid binding and transfer protein, GM2AP, to form GM3, which is degraded to lactosylceramide by an α-sialidase with the help of Sap B [49]. GM2AP and Sap B are lipid-binding and lipid-transfer glycoproteins. Their inherited defects cause mainly GG GM2 storage in AB-variant of GM2-gangliosidosis, and sulfatide accumulation in a rare juvenile form of metachromatic leukodystrophy, respectively. Sap C and D are lysosomal lipid binding and vesicle fusion glycoproteins. Genetic defects of Sap C attenuates glucosylceramide catabolism in a rare form of juvenile Gaucher disease, whereas Sap D deficiencies trigger the lysosomal accumulation of hydroxylated, long chain ceramides in a rare form of Farber disease [8]. The hydrolysis of glucosylceramide by β-glucocerebrosidase in the presence of cholesterol [50] can also lead to the formation of β-glucosylcholesterol by transglucosylation [51], thereby raising lysosomal levels of glucosylcholesterol.

In mice, the major catabolic pathway of GM1 and GM2 is the same as in humans, but can be bypassed. A sialidase slowly removes sialic acid from the monosialogangliosides GM1 and GM2, generating the respective asialo-derivatives, GA1 and GA2, which are further catabolized by lysosomal hexosaminidases and β-galactosidases. Therefore, Hex A deficient mice avoid a major glycolipid accumulation and are a poor model of TSD [9,35]. They keep a slow GM2 turnover and generate only a minor GM2 accumulation, especially in Purkinje and Pyramidal cells, allowing an almost normal life span [35]. Finally, GM3, GA1, and GA2 are catabolized further in the lysosomal system to release their building blocks, monosaccharides, free fatty acids, and sphingoid bases as reviewed recently [9].

### Maturation of ILVs and Regulation of GG Catabolism at ILV Surfaces

GG and other amphiphilic sphingolipids are membrane components of eukaryotic cells. Due to their amphiphilic nature, their insolubility in aqueous solutions, and based on experimental evidence obtained in vitro and by in vivo studies using murine and human cell cultures, we assume that the surface of ILVs, carrying GGs and other complex lipids, is the main location of their catabolism in the lysosomal compartment [8,9].

The lysosomal lumen contains more than 70 different hydrolases, including lipases, phospholipases, phosphatases, glycosidases, sulfatases, and nucleases, besides lipid binding SAPs and many other proteins of unknown function [9,52]. They are soluble glycoproteins being active at low pH-values, which prevail in healthy and active lysosomes. At low pH-values, most lysosomal hydrolases are protonated and positively charged, whereas the surfaces of the ILV membranes are negatively charged, mainly due to their high content of the anionic lysolipid bis(monoacylglycero)phosphate (BMP) and the possible presence of other anionic phospholipids in the ILV membranes [10]. BMP is an intermediate of the phosphatidylglycerol catabolism and can enrich in ILV membranes to reach 40–60 mol% of their phospholipid content [8,53,54], mainly due to its slow catabolism. It can generate a negative zeta potential on the surfaces of ILVs [10], which electrostatically attracts positively charged hydrolases and SAPs to the sphingolipid-substrate carrying ILV-membranes, speeding up their catabolic rates [8,55,56] (Figure 1).

For diagnosis of LSDs, most lysosomal hydrolases are usually assayed in vitro with the help of synthetic and soluble fluorogenic substrates [57,58]. These convenient assays are an easy way to detect the presence of a lysosomal hydrolase in patients’ samples and to determine its activity in vitro. The activity measured in vitro with soluble synthetic substrates, however, does not indicate, in any way, the level of the sphingolipid-substrate cleaving activity of the patient’s hydrolase in vivo [6], since the sphingolipid cleaving activity of a lysosomal hydrolase can be strongly regulated and modified in vivo by genetic and by post translational modifiers which do not affect its activity against soluble substrates in vitro.

**Figure 3:**
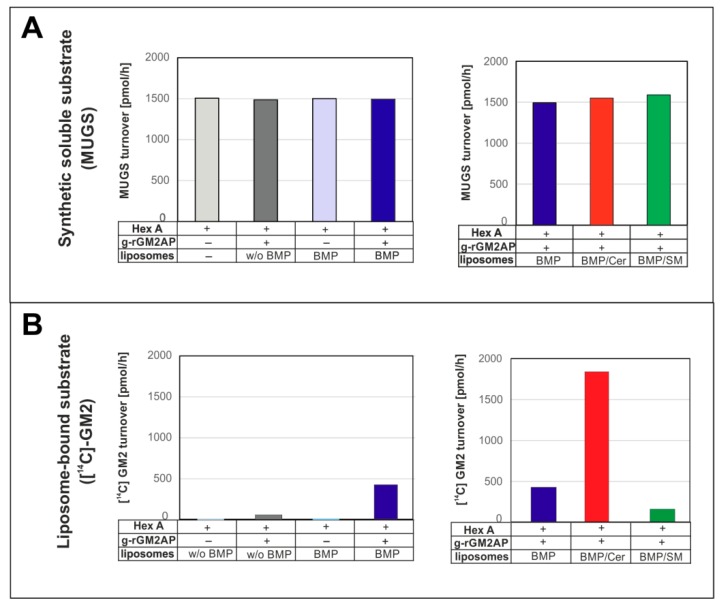
(**A**) Membrane lipids and GM2AP do not affect the hydrolysis of artificial, water-soluble substrate fluorogenic 4-methylumbelliferyl-6-sulfo-2-acetamido-2-deoxy-β-d-glycopyranoside (MUGS) by Hex A but (**B**) they strongly affect and regulate the catabolism of liposome-bound radiolabeled native GM2, reflecting the in vivo conditions at the ILVs. This figure is modified from [6].

As genetic modifiers we consider the SAPs, small lipid-binding, lipid-transfer and/or vesicle-fusion glycoproteins of the lysosomal compartment. These cofactors are essential for the glycosphingolipid and sphingolipid cleaving activity of lysosomal hydrolases to reach physiologically relevant levels. Their inherited deficiencies can cause fatal storage diseases [8,9,59] despite the presence of fully active hydrolases, detectable with soluble fluorogenic substrates in patient’s cultivated cells or blood samples. As posttranslational modifiers of the lipid-cleaving activity of lysosomal hydrolases we consider the strong inhibitory action of chondroitin sulfate (accumulating in the lysosomes of Hurler, Hunter, Sanfilippo, and Sly disease [7]) on the GM2 catabolism, as well as the collective properties of the sphingolipid-substrate carrying vesicle-membranes (e.g., extent of negative surface charge of ILVs to attract and bind protonated and positively charged hydrolases and the lipid composition of the vesicular membranes, especially the presence of stimulatory (e.g., BMP, ceramide) or inhibitory lipids (cholesterol, sphingomyelin)). These genetic and posttranslational modifiers strongly regulate the lipid-cleaving activity of lysosomal hydrolases, but rarely affect their activity against soluble, synthetic substrates used in vitro to diagnose lysosomal lipid storage diseases, as recently detailed in reconstitution experiments for the regulation of GM2 cleavage by Hex A in comparison to the unaffected cleavage of the soluble substrate MUGS (4-methylumbelliferyl-6-sulfo-2-acetamido-2-deoxy-β-d-glycopyranoside) [6] (Figure 3).

Organellar membranes of eukaryotic cells maintain an organelle specific protein and lipid composition [60]. Cellular plasma membranes are rich in stabilizing lipids like cholesterol (up to 40 mol% of their lipid content), and in their outer leaflet they maintain high levels of sphingomyelin and complex glycosphingolipids. Both, cholesterol and sphingomyelin, were identified as major inhibitors of key steps of lysosomal sphingolipid catabolism [8,9]. Therefore, the conversion of inhibitory sphingomyelin into stimulatory ceramide by acid sphingomyelinase (ASM) along the endocytose pathway at the level of late endosomes, and the removal of inhibitory cholesterol from nascent ILVs by two sterol-binding and -transfer proteins, Niemann–Pick disease protein C type 1 (NPC1) and NPC2, are essential to allow a physiological sphingolipid and GSL turnover [10,38,61,62].

In LSDs, disturbances of lipid sorting as it may occur during endocytosis and progressive accumulation of storage material can cause a dysregulation of lysosomal lipid catabolism. Indeed, primary sphingomyelin accumulation in Niemann–Pick disease types A and B triggers a secondary increase of the cholesterol levels by inhibiting the NPC2-mediated cholesterol export from ILVs [10,63]. Both storage compounds cause a mild accumulation of GGs, like GM2 and GM3 [63,64], and a secondary rise of glycolipids like glucosylceramide in the lysosomal compartment by affecting their catabolic pathways directly. Increasing lysosomal cholesterol levels in Niemann–Pick disease type C inhibit the activity of several SAPs studied so far, the GM2AP, Sap A and Sap B [6,38,65,66], presumably also attenuating the lysosomal turnover of sphingolipids (e.g., sulfatides, lactosylceramide, GG GM1, sphingomyelin, globotriaosyl- and globotetraosylceramide, etc.).

## 4. Cascading Errors in LSDs

Defective lysosomal hydrolases attenuate or even block the catabolism of their substrates and trigger their accumulation, often causing a fatal disease. This basic concept, however, does not explain, why cholesterol and GSLs accumulate in various LSDs that do not suffer from a deficiency of their specific secretory or catabolic machinery [67].

Lysosomal degradation of complex lipids is regulated by molecules in the microenvironment of the reaction and is often inhibited by progressively increasing levels of primary storage compounds [6,7,8], as discussed above, or by chronically added cationic amphiphilic drugs (CADs) [68]. For example, high sphingomyelin levels in the lysosomes of Niemann–Pick diseases types A and B inhibit the cholesterol secretion from the lysosomal compartment and generate a massive secondary lysosomal cholesterol storage [63]. Following the cascade model, both storage lipids trigger themselves an additional, a tertiary lysosomal accumulation of GGs and GSLs in type A and B of Niemann–Pick disease by affecting their catabolic pathways, as outlined above. That means, metabolic modifiers within the lysosomal compartment may dysregulate cellular metabolism which includes also the accumulating material in LSDs, for instance lipids like sphingomyelin and cholesterol, cationic bases, sphingosine, sphinganine, and toxic lysosphingolipids like glucosylsphingosine [69], galactosylsphingosine, and others, as well as the primary storage compound chondroitin sulfate in MPS (Hunter, Hurler, Sanfilippo, and Sly syndrome) [7]. These accumulating modifiers often inhibit genetically unaffected catabolic pathways strongly in the lysosomal system. If one compound (a lipid or a mucopolysaccharide) is stored and interferes with another catabolic lipid pathway, then the latter will also start to accumulate its substrates as secondary storage compounds. They can dysregulate further catabolic pathways in the lysosome, affect the cellular metabolism and may trigger serious pathological consequences for the patients.

Secondary storage compounds originating by a cascade of errors like neuronal accumulation of GM2 in Niemann–Pick disease type C and in MPS like Hunter, Hurler, Sanfilippo, and Sly syndrome, can be neurologically debilitating.

## 5. Lysosomal Storage Disorders with Secondary Ganglioside Accumulation

Lysosomal GG and GSL accumulation has been observed in LSDs without a genetic defect in the GG catabolism, e.g., in Niemann–Pick diseases, some MPSs [64,70], (glyco)sphingolipidoses, prosaposin deficiency, mucolipidoses (MLs), glycoproteinoses, neuronal ceroid lipofuscinoses (NCLs), and hereditary spastic paraplegia (HSP) (Table 2). In this case, mainly the GGs, GM2 and GM3, are accumulated. They are minor compounds (1–2% of the total gangliosides) of the human brain and their proportion is even smaller in mice. A secondary accumulation of GM2 and GM3, however, is associated with neuropathology in many LSDs. In the literature, their secondary accumulation is often based on the general assumption of a nonspecifically disturbed lysosomal catabolism without presenting any molecular mechanism.

**Figure 4:**
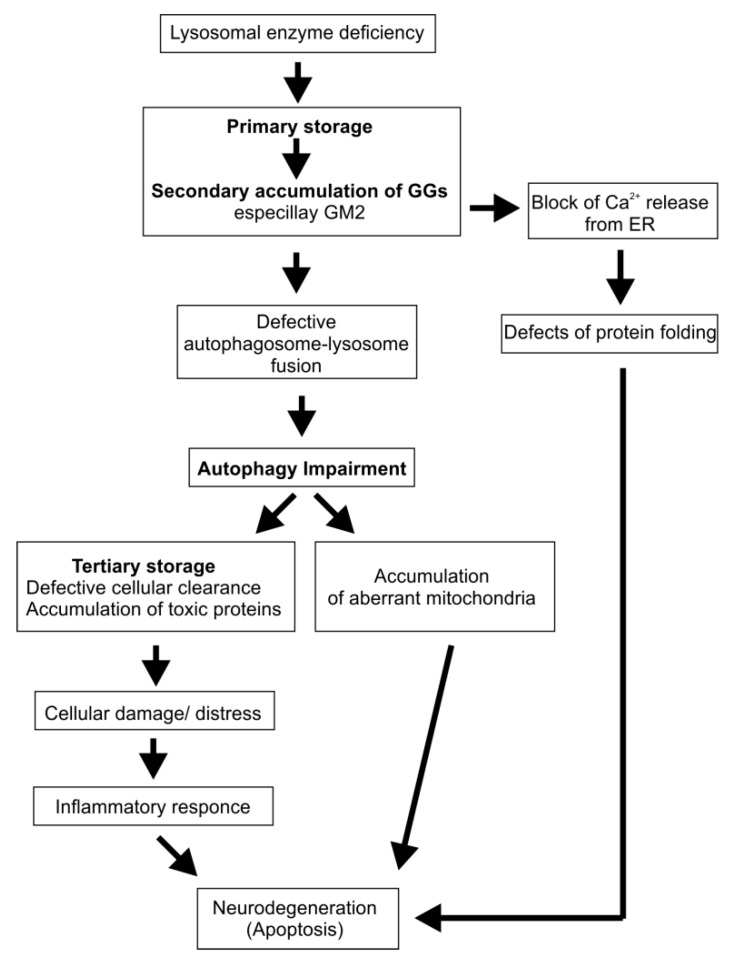
Proposed model for the pathogenesis in lysosomal storage diseases (LSDs). The model is modified after [77].

Storage compounds primarily localize to the endolysosomal compartment. However, in some LSDs an accumulation in other cellular membranes besides the endolysosomal system was also observed [71,72]. This can be mediated by membrane-flow from endolysosomal to other cellular membranes, or by transfer at membrane contact sites, or even by protein transport [73,74,75]. Lysosomal accumulation of metabolites can affect several functions of the organelle e.g., autophagy [76], Ca^2+^-homeostasis [77,78,79], and signaling cascades [80]. A proposed model for impaired autophagy and neurodegeneration in LSDs is given in Figure 4.

### 5.1. Sphingolipidoses

#### 5.1.1. Niemann–Pick Disease Type A and B

The pediatrician Albert Niemann described the first patient with infantile Niemann–Pick disease in 1914, an Ashkenazi Jewish infant with massive and progressive hepatosplenomegaly and neurodegeneration, who died at 18 months of age. In her reticuloendothelial cells, Niemann observed a massive lipid-storage at autopsy [81]. Sphingomyelin was identified by Ernst Klenk as being the main storage compound [82]. An inherited deficiency of the sphingomyelin-cleaving hydrolase, ASM (encode by *SMPD1*), causes the phospholipid accumulation in Niemann–Pick disease patients [83]. Purification of the secreted enzyme from human urine to homogeneity allowed the identification of the human ASM as an unspecific lysosomal phospholipase C [10,84]. ASM is a glycoprotein using five *N*-glycosylation sites [85].

Its precursor protein is synthesized at the endoplasmic reticulum to generate functional secretory and lysosomal molecular forms of the ASM by proteolytic processing and trimming of its glycan chains [86]. Sequence analysis allowed the detection and characterization of the human ASM coding cDNA [87] and the identification of the first Niemann–Pick disease causing mutations [88,89], which induce a massive lysosomal sphingomyelin accumulation in Niemann–Pick disease type A and type B [83]. Brain extracts of Niemann–Pick disease type A patients contain increased levels of glucosylceramide, dihexoside, and trihexoside, as well as GM2 [90] and GM3 (Table 2).

The primary storage compound sphingomyelin is a strong inhibitor of lysosomal cholesterol secretion by affecting the late endosomal and lysosomal steroid transfer protein NPC2 [10], thereby triggering an almost equimolar accumulation of cholesterol in the lysosomal system [63]. Both storage compounds, sphingomyelin and cholesterol as well as chondroitin sulfate, accumulating in some MPS, are effective inhibitors of several catabolic pathways of GGs and GSLs, inducing a secondary lysosomal accumulation of small GGs and GSLs in Niemann–Pick diseases and some MPSs [6,7,8,9].

There is currently no cure for patients with Niemann–Pick disease type A or B. Few people with type B were treated by bone marrow transplantation [91]. The development of enzyme replacement therapy with ASM (in clinical trial, [92]) and gene therapies could be helpful for the treatment of type B patients. Recently, Samaranch et al. published a successful study of the treatment of animal models of Niemann–Pick disease type A by adeno-associated viral vector serotype 9-based gene therapy [93].

#### 5.1.2. Niemann–Pick Disease Type C

Niemann–Pick disease type C is a fatal, mostly juvenile and later onset disease, primarily caused by a lysosomal cholesterol accumulation, induced by genetic defects in the cholesterol secretion from the lysosomal compartment [94]. Cholesterol secretion is mainly achieved by two nonredundant functionally cooperating steroid transport proteins, NPC1 and NPC2 [95]. Inherited defects in NPC1 [96] and NPC2 [97] cause a progressive lysosomal accumulation of cholesterol, that apparently triggers a secondary accumulation of lipids, despite the “fact”, that no genetic defect has been observed in their catabolism. The type C of Niemann–Pick disease has almost normal ASM levels and a complex lipid storage pattern in liver and spleen with moderate increases of up to five-fold in unesterified cholesterol, sphingomyelin, BMP, as well as smaller amounts of other phospholipids and glycolipids [70]. In the brain, however, most abnormalities are accumulations of glycolipids like glucosylceramide, lactosylceramide, little GGs, like GM2 and GM3, and small amounts of cytotoxic sphingoid bases, sphingosine and sphinganine [98]. Purkinje cells of the mouse model of Niemann–Pick disease type C accumulate GM2 but no GM3 [95].

At the posttranslational level, cholesterol is a potent inhibitor of all SAPs studied so far, Sap A, Sap B and GM2AP [38,65,66]. An impaired activity of the SAPs may well favor a lysosomal accumulation of GM2, GM3, and lactosylceramide [99]. For the additional accumulation of toxic and cationic sphingoid bases like sphingosine and sphinganine, however, no mechanistic explanation is known at the molecular level. It is likely, however, that their secretion from the lysosomal compartment is impaired by increasing levels of the primary storage compound, cholesterol. Like other cationic amphiphiles [10,56,100], cationic sphingoid bases may compensate the negative surface charge of intraendolysosomal luminal vesicles, release positively charged ASM from their surfaces and trigger its proteolytical digestion within the late endosome and lysosome [10,56,100], thereby elevating lysosomal sphingomyelin levels progressively. Indeed, feeding both, sphingomyelin and cholesterol, to cultured cells, lowers their ASM levels [101]. Increasing levels of cationic sphingoid bases may also contribute indirectly to the secondary sphingomyelin accumulation in Niemann–Pick disease type C and may inhibit also cleavage of other phospholipids such as phosphatidylglycerol, and phosphatidylcholine by ASM [10,84]. Accumulating sphingomyelin can also inhibit lysosomal Ca^2+^ release by affecting the principle Ca^2+^ channel TRPML1 (TRPML is an acronym for transient receptor potential cation channel, mucolipin subfamily) in the lysosomes and thereby contribute to neurodegeneration [102].

The laboratory diagnosis of Niemann–Pick disease is difficult, and meaningful genetic analysis is expensive and takes a long time. More rapid is the mass spectrometry-based analysis of potential biomarkers (reviewed in [103]). They are useful for a screening, but not very specific and can lead to false positive results. Currently, lyso-sphingomyelin-509 (LysoSM-509) is used for the primary diagnosis of Niemann–Pick disease type C [104]. Another biomarker is cholestane-3β,5α,6β-triol [105]. Both biomarkers, however, also accumulate in Niemann–Pick disease types A and B [103]. As a therapeutic approach, the drug miglustat (Zavesca) is used for patients with mild to moderate symptoms of type C.

#### 5.1.3. Gaucher Disease

Gaucher disease is one of the most common lysosomal storage diseases and it is caused by a deficiency of acid glucosylceramide-β-glucosidase (EC 3.2.1.45, also known as lysosomal glucosylceramidase or β-glucocerebrosidase, GBA1) resulting in a primary accumulation of glucosylceramide and the toxic amphiphile glucosylsphingosine [106,107,108]. This disorder is divided into three clinical subtypes (Type I: Adult form with non-neuropathic impairment, which is the most common form of the disease in western countries; Type II: A rare and acute form involving neurological abnormalities; type III: is an intermediate variant between types 1 and 2). The complete deficiency of the GBA1 activity leads to a perinatal fatal form, the “collodion baby” phenotype with a severe impairment of barrier functions in the skin [109,110].

The overall GG concentration in brain specimens of Gaucher patients seems to be normal, whereas an increased proportion of minor GGs, GM2 and GM3, has been reported [106]. GM3 is strikingly elevated in plasma of most Gaucher type I patients comparable to that of glucosylceramide, the primary storage lipid [111]. Elevated GM3 levels may play a role in the insulin resistance of the Gaucher patients. However, in the postmortem nervous tissue of a Gaucher type II patient ganglioside levels were reduced with a relative increase of GD3 [112].

Several biomarkers have been investigated for GD, however, none of them is perfect. Chitotriosidase activity levels have been considered as a classic biomarker [105]. They are used as an indicator of disease severity and its response to therapy. Currently, glucosylsphingosine (lyso-Gb1) is used as a more effective alternative to chitotriosidase and CCL18 [113,114].

Two specific types of treatment are available mainly for adult GD patients: enzyme replacement therapy (ERT) by β-glucocerebrosidase (Imiglucerase or Velaglucerase) and substrate reduction therapy (SRT) by Miglustat or Eliglustat.

#### 5.1.4. Krabbe Disease

Krabbe disease or globoid cell leukodystrophy, is an autosomal recessive disorder caused by a deficiency of β-galactocerebrosidase (EC 3.1.6.8), the lysosomal enzyme responsible for the degradation of the myelin lipid galactocerebroside to ceramide and galactose. This LSD is characterized by major pathological changes like an extensive demyelination, gliosis, and appearance of storing macrophages (globoid cells) in the white matter.

The ganglioside distribution of cerebral cortex and white matter of children, who had died with Krabbe’s disease, was severely altered. Cerebral cortex and white matter had reduced levels of GD1a and GM1, while levels of minor GGs, GD2, GD3, and GM3, were strongly increased [115].

For diagnosis and treatment of Krabbe disease, psychosine (galactosylsphingosine) analysis is applied as a marker in a blood test [116]. Hematopoietic stem cell transplantation serves as a therapeutic approach of Krabbe disease. It is slowing disease progression. Further patient treatments are reviewed in [117].

#### 5.1.5. Metachromatic Leukodystrophy (MLD)

MLD is a rare hereditary LSD caused by deficiency of arylsulfatase A (EC 3.1.6.8) or of the Sap B. Arylsulfatase A removes the sulfate residue from sulfatide (cerebroside sulfate) in the presence of Sap B. Arylsulfatase A-deficient cells primarily accumulate the anionic glycosphingolipid sulfatide, which is a major component of myelin. It is crucially involved in myelin formation and cell-to-cell interactions. Enhanced sulfatide levels were found in many tissues of the body (nervous system, kidney, testes, and other organs) [118]. Several gangliosides, especially the minor ones, GM2, GD3, GM3, and GD2, have been reported elevated in the white matter of patients suffering from various leukodystrophies, including MLD [119]. Only a two-fold GM2 accumulation was observed in a mice model of MLD [120].

Researchers are currently looking for suitable biomarkers in the blood (plasma) of MLD patients. Glycosylsphingosin-sulfatide (lyso-Gb1-sulfatide) has been identified so far as a sensitive and specific biomarker. An overview for different therapeutic approaches is given in [121].

#### 5.1.6. Farber Disease

Inherited functional defects of acid ceramidase (EC 3.5.1.23, an *N*-acylsphingosine amidohydrolase) leads to an accumulation of ceramides in Farber disease. The enzyme catalyzes the catabolism of ceramide to sphingosine and fatty acid. In vivo, the enzyme activity is stimulated by Sap D [122]. Patients with Farber disease develop a severe lipogranulomas with subcutaneous nodules, painful and progressive joint deformations, and progressive hoarseness. A moderate nervous dysfunction is related to the primary storage of ceramides, preferentially containing long chain fatty acids and a secondary accumulation of gangliosides in neurons and anterior horns cells of the spinal cord [123,124].

Diagnosis of Farber disease is based on the activity level of acid ceramidase in peripheral blood leukocytes, cultured lymphoid cells or skin fibroblasts. Screening for biomarkers identified C26- ceramide as a potential candidate for this disorder [125]. Currently, there is no effective therapy available for Farber patients. Enzyme replacement therapy has recently been carried out at an experimental level in cultured cells and Farber mice [126].

#### 5.1.7. Prosaposin Deficiency

Prosaposin is the precursor protein of four saposins designated Sap A–D. They are generated by proteolysis of prosaposin in late endosomes and in lysosomes. Saposins (and the GM2AP) are essential for the degradation of GSLs with short oligosaccharide chains [8,9]. Patients and corresponding mouse models deficient in prosaposin develop a massive accumulation of intralysosomal luminal storage vesicles and membranes accompanied by a progressive accumulation of undegraded sphingolipids, including ceramide, glucosylceramide, galactosylceramide, lactosylceramide, digalactosylceramide, sulfatides, GG GM3, and globotriaosylceramide [127]. It is not clear if the observed GG accumulation is a primary or secondary effect of the prosaposin deficiency.

### 5.2. Mucopolysaccharidoses (MPSs)

MPSs are a group of rare LSDs caused by inherited deficiencies of eleven different enzymes degrading glycosaminoglycans like dermatan sulfate, heparan sulfate, keratan sulfate, chondroitin sulfate, or hyaluronan. They include seven distinct subgroups (I, II, III, IV, VI, VII, and IX) (Table 2) [5]. Metabolites of defective steps in glycosaminoglycan catabolism are stored in the endolysosomal system of cells in many different tissues including the brain and are in part excreted in urine. Furthermore, a secondary accumulation of minor GGs, GM2 and GM3, was detected in the brain of several MPS patients or MPS mice models [128].

Many approaches use the analysis of glycosaminoglycan fragments by mass spectrometry for the diagnosis for MPS [129,130]. New quantitative mass spectrometry methods also allow the direct detection of dermatan sulfate, heparan sulfate, and chondroitin sulfate in urine and cerebrospinal fluid and facilitate the diagnosis for patients with MPS I, II, III, IVA, and VI [131].

#### 5.2.1. MPS I (Hurler Syndrome)

MPS I (Hurler Syndrome) is caused by a deficiency of α-L-iduronidase (E.C. 3.2.1.76), catabolizing glycosaminoglycans carrying a terminal α-iduronic acid residue. The loss of α-L-iduronidase activity triggers mainly a progressive storage of dermatan sulfate and heparan sulfate, accompanied by a secondary accumulation of GGs, GM2 (by inhibition of its catabolism, see above) and GM3, markedly in white and grey matter of the brain in patients with Hurler syndrome [128].

Early diagnosis of MPS I allows an approach of intravenous enzyme replacement therapy (Iduronidase sold as Aldurazyme (Genzyme)), which provides stabilization of the clinical symptomatology [132]. Another therapeutic approach is hematopoietic stem cell transplantation.

#### 5.2.2. MPS II (Hunter Syndrome)

MPS II (Hunter Syndrome) is an X-linked recessive disorder preferentially affecting male patients. It is caused by a deficiency of the lysosomal enzyme iduronate-2-sulfatase (EC 3.1.6.13). The lack of its enzyme activity causes mainly storage of heparan sulfate and dermatan sulfate in all body tissues and leads indirectly to a secondary accumulation of GM2 (by inhibition of its catabolism, see above) and GM3 [128,133].

MPS II patients suffer pulmonary dysfunction, skeletal deformities, cardiomyopathy and, in most patients, neurological decline. For boys with milder Hunter syndromes, the enzyme replacement therapy can help to slow disease progression [134].

#### 5.2.3. MPS III (Sanfilippo Syndrome)

MPS III, also known as Sanfilippo syndrome, is a rare autosomal recessive LSD that primarily affects the brain and spinal cord. Mutations in four different genes can lead to Sanfilippo syndrome (A: *SGSH* encodes heparan-*N*-sulfatase (EC 3.10.1.1), B: *NAGLU* encodes α-*N*-acetylglucosaminidase (EC 3.2.1.50), C: *HGSNAT* encodes acetyl-CoA:α-glucosaminide *N*-acetyltransferase (EC 2.3.1.78), and D: *GNS* encodes *N*-acetylglucosamine-6-sulfatase (EC 3.1.6.1)). Aside from the primary storage of heparan sulfate also GGs, GM2 (by inhibition of its catabolism, see above), GM3, GD2, are accumulated secondarily in patients with Sanfilippo Syndrome or their mice models [128,135,136].

There is no cure for MPS III. Recent studies represent interesting approaches for enzyme replacement therapy, gene therapy, and substrate reduction therapy (reviewed in [137]).

#### 5.2.4. MPS VI (Maroteaux–Lamy Syndrome)

MPS VI or Maroteaux–Lamy syndrome is caused by deficiency of arylsulfatase B (*N*-acetylgalactoamine-4-sulfatase, EC 3.1.6.12). The lysosomal hydrolase splits sulfate esters off glycosaminoglycans, mainly dermatan sulfate and heparan sulfate [5]. Complete or partial lack of arylsulfatase B activity leads to an accumulation of dermatan sulfate. Studies on fibroblasts of MPS VI patients showed an impaired autophagy with an accumulation of polyubiquitinated proteins and mitochondrial dysfunction [138,139]. Also an increase of GM2 and GM3 levels has been detected [140].

There is no cure for MPS VI, but intravenous enzyme replacement therapy by galsulfase (Naglazyme) [141] may ameliorate certain somatic symptoms, however, does not reduce neurological symptoms.

#### 5.2.5. MPS VII (Sly Syndrome)

Sly syndrome, also called MPS VII is a very rare autosomal recessive LSD due to a β-glucuronidase (EC 3.2.1.31) deficiency. It shows a wide range of severity and system heterogeneity similar to MPS I and II [5]. Patients exhibit a primary accumulation of heparan sulfate, chondroitin sulfate, and dermatan sulfate and, as a secondary effect, a storage of GM2 (by inhibition of its catabolism, see above) and GM3 [5,142].

### 5.3. Mucolipidoses (MLs)

MLs are a group of rare autosomal recessive diseases dominated by an accumulation of soluble oligosaccharides. They include sialidosis (ML I), I-cell disease (ML ΙΙ), pseudo-Hurler polydystrophy (ML III), and ML IV. However, sialidosis is now classified as a glycoproteinosis and ML IV as a gangliosidosis.

#### 5.3.1. Mucolipidosis ΙΙ (I-cell Disease) and Mucolipidosis ΙIΙ (Pseudo-Hurler Polydystrophy)

Mucolipidosis types II and III (ML II and ML III) result from a deficiency of the enzyme *N*-acetylglucosamine-1-phosphotransferase (EC 3.1.4.45), which phosphorylates target carbohydrate residues on *N*-linked glycoproteins. This enzyme is responsible for synthesis of the mannose-6-phosphate recognition marker that is essential for lysosomal enzyme targeting. Without this phosphorylation, the glycoproteins are not destined for lysosomes, and they escape by the fault pathway to the outside of the cell. A moderate GM1 accumulation is observed in these LSDs [140,143]

The symptoms of ML II and ML III are similar to but more severe than those of Hurler syndrome. At this time there is no treatment available.

#### 5.3.2. Mucolipidosis ΙV (Mucolipidin 1 Deficiency)

The disorder is caused by mutations in the *MCOLN1* gene, which encodes mucolipin 1, a non-selective cation channel. The channel is an integral membrane protein with homology to non-selective cation channels including the transient receptor potential channels (TRPML). Mutations in *MCOLN1* disrupt many cellular functions and cause neurodevelopmental disorders by unknown mechanisms. Mucolipin1 is involved in the regulation of fusion/fission of vesicles along the endocytic pathway and in some aspects of the lysosomal Ca^2+^ homeostasis [144,145]. Biochemical studies indicate that ML IV-patients suffer from a deficient sialidase activity hydrolyzing gangliosides GM3 and GDla, and an increased urinary excretion of glycolipids and phospholipids [146,147].

### 5.4. Glycoproteinoses

#### 5.4.1. Galactosialidosis

Galactosialidosis is a glycoprotein storage disease caused by an inherited deficiency of the lysosomal protective protein/cathepsin A (PPCA) which is associated in a complex with both, α-neuraminidase (sialidase, NEU1, EC 3.2.1.18) and acid β-galatosidase (β-Gal, EC 3.2.1.23). PPCA is a multifunctional enzyme which binds and protects these two glycosidases from premature proteolysis. Therefore, the loss of PPCA results in a deficiency of α-neuraminidase and β-galactosidase activities [148].

The deficiency of these enzymes leads to an accumulation of sialyloligosaccharides in lysosomes and in excreted body fluids. Furthermore, juvenile and adult galactosialidosis patients featured an accumulation of GM1, GD1a, GM2, and GM3 in sympathetic and spinal ganglia and in gray matter of the spinal cord [149,150]. Due to the GG accumulation galactosialidosis is often classified as a glycosphingolipidosis. The storage of GM3, GD1a, and GM1 could be explained by the loss of α-neuraminidase and β-galactosidase, respectively [149], however, neither of these defects appears to account for the increase in GM2.

#### 5.4.2. α-Mannosidosis

α-Mannosidosis affects the lysosomal glycoprotein catabolism and is caused by inherited deficiencies of the α-mannosidase activity (EC 3.2.1.24), leading to the a progressive storage of undegraded mannose-rich oligosaccharides in many tissues including brain and viscera [151]. The disease was first described in 1967, by the Swedish physician Okerman.

In α-mannosidosis all neurons exhibit a storage of water-soluble oligosaccharides, whereas a secondary accumulation of minor GGs, GM2 and GM3, was observed only in scattered numbers of pyramidal and GABAergic neurons of the cerebral cortex [152]. Animal models of the disease also accumulate the minor GGs, GM2 and GM3 in brain, while total ganglioside levels appear to be in the normal range [32].

α-Mannosidosis could possibly be treated by bone marrow transplantation or enzyme replacement therapy (Lamzede) [153,154].

#### 5.4.3. Sialidosis

Sialidosis is characterized by accumulation of sialic acid-containing compounds (mainly sialyloligosaccharides and sialoglycoproteins) in cells caused by a functional loss of lysosomal NEU1 (sialidase, acid neuraminidase, EC 3.2.1.18). Certain glycolipid levels (GM3, GD3, GM4, and LM1) are elevated in visceral organs, but not in brain. Whereas GM3 is a substrate of NEU1 in vitro [49] and therefore presumably a primary storage compound, it remains unclear, if NEU1 also acts on all the other accumulating gangliosides.

Patients with a deficiency of lysosomal NEU1 exhibit progressive deterioration of muscle and central nervous system functions. No cure or specific therapies are currently available.

### 5.5. Neuronal Ceroid Lipofuscinoses (NCLs)

The NCLs, collectively also called, Batten disease, are a group of now 14 autosomal recessively inherited neurodegenerative LSDs due to the excessive lysosomal accumulation of neuronal and extraneuronal autofluorescent lipopigments (also called ceroid) with characteristic ultrastructural features [155]. Clinically, NCLs are characterized by progressive decline of cognitive and motor function, progressive cerebellar atrophy, retinopathy, myoclonic epilepsy and early death [155]. Mutations in 14 different genes (called *CLN*s) led to various forms of NLCs. The mechanisms causing neurodegeneration in different NCLs are poorly understood. It is proposed that there is a correlation between storage material and neurodegeneration [156,157].

A secondary GG accumulation is found in NCL 3, NCL 6, and NCL 10, the congenital cathepsin D deficiency).

#### 5.5.1. NCL 3 (Batten Disease)

NCL 3 is a fatal disease of the nervous system that is caused by mutations in the CLN3 gene, which cause a lipofuscin (ATPase subunit c) accumulation. CLN3 appears to be a multifunctional protein involved in the regulation of lysosomal acidification, lysosomal arginine import, apoptosis, and vesicular membrane traffic [158].

In the corresponding mouse model, the levels of cellular gangliosides, particularly GM3, GM2, GM1a, and GD1a were investigated. The levels of GM1a and GD1a were significantly reduced, whereas a highly significant increase in GM3 could be detected [159].

The first symptoms of NCL 3 typically begin in childhood. After 4–6 years of normal development, patients show vision impairment, intellectual disability, movement problems, speech difficulties, and seizures. Until now, no specific treatment is known that can stop or reverse the symptoms of NCL 3.

#### 5.5.2. NCL 6

The function of the highly conserved protein, encoded by the *CLN6* gene, is unknown. Mutations of the *CLN6* gene cause NCL 6 disease, which primarily affects the nervous system and can be diagnosed in early and late childhood. Patients with NCL 6 show a loss of previously acquired skills. Further symptoms are epilepsy, ataxia, muscle twitches, impaired speech, and vision loss. *Cln6*-deficient mice store autophagosome/autolysosome-like bodies in neuronal perikarya [160] accompanied by increasing amounts of GM2 and GM3 [156].

#### 5.5.3. NCL 10 (Congenital Cathepsin D deficiency)

NCL 10 (CLN10 disease) belongs to a group of severe diseases that primarily affect the nervous system. Individuals with this condition typically show signs and symptoms soon after birth. NCL 10 disease is caused by mutations in the *CTSD* gene which encodes cathepsin D. Cathepsin D is a major lysosomal endopeptidase, which is critical in the degradation of long-lived proteins.

*Ctsd*-deficent mice have highly elevated levels of BMP [156] and storage of autophagosome/autolysosome-like bodies in neuronal perikarya [160]. Granular osmiophilic deposits are associated with an accumulation of SAPs, especially Sap A [161]. Furthermore, GM2 accumulates in neurons whereas glia primarily harbor GM3 storage [156].

### 5.6. Hereditary Spastic Paraplegia (HSP)

HSP is clinically and genetically a heterogeneous group of neurodegenerative disorders that are clinically characterized by progressive weakness and spasticity of the legs. These symptoms are caused by the degradation of the upper motor axons in the corticospinal tracts [162]. HSP is due to mutations in over 70 genes [163]. Most of them encode proteins involved in membrane traffic and modeling, endosomal tubule fission, and lysosomal biogenesis and function [164].

#### Hereditary Spastic Paraplegia Caused by Mutations in the AP 5/SPG11/SPG15 Complex

The adaptor proteins (APs) are a family of five heterotetrameric complexes with important functions in vesicle trafficking of molecules from one subcellular location to another [165]. These complexes concentrate the correct cargo molecules in vesicles that bud or extrude off of one organelle and travel to another location, where the cargo is delivered. The recently identified AP 5 complex is localizes at the endolysosomal compartment.

It has been shown that two other proteins, spatacsin (SPG11) (SPG is an acronym for spastic paraplegia gene) and spastizin (SPG15) formed a stable complex with AP 5 [166]. Mutations in the ζ-subunit of AP 5 complex (SPG48), encoded by the *AP5Z1* gene, as well as in the associated proteins spatacsin (SPG11 or called KIAA1840) and spastizin (SPG15 or called ZFYVE26) lead to an accumulation of aberrant endolysosomes filled with undigested material, and highlight the role of endolysosomal dysfunction in the pathology of HSP and other neurodegenerative disorders [167,168,169]. Hirst et al. found that the loss of AP 5 leads to defects in the retrieval of several proteins from the late endosomes back to the Golgi apparatus [170] which results in a endolysosomal dysfunction.

In the brain of patients with a mutation in *SPG11* gene and in *Spg11* knockout mice a lipid accumulation especially of GM2, GM3, GD2, and GD3 as well as an accumulation of autophagy markers (e.g., p62) were observed in the endolysosomes [171,172]. Unfortunately, no studies exist about the lipid content of AP 5 or SPG15 deficient cells or tissue. However, we presume that a GG accumulation could be found also in endolysomes of AP 5 and SPG15 deficient cells.

Furthermore, abnormal lysosomes are also found in other forms of HSP. The lysosomal dysfunction can be caused by dysfunctional spastin (SPG4), encoded by *SPAST* gene, or strumpellin (SPG8), encoded by *KIAA0196* gene, or SPG31 (REEP1) [164].

### 5.7. TgCRND8—An Alzheimer’s Disease Mouse Model

In Alzheimer brains and in Alzheimer disease mouse models, the levels of major gangliosides (e.g., GM1, GD1a, GD2b, and GT1b) appear to be mostly unchanged [173], whereas levels of minor GGs such as GM3 are often increased [173,174,175,176]. It could be expected, however, that the increasing loss of the ganglioside rich nerve endings during the progression of the disease could trigger a decrease of the ganglioside levels, at least in the grey matter.

An Alzheimer model, the TgCRND8 mouse, overexpresses the human amyloid precursor protein (APP) and exhibits autophagy-related pathology in neurons with enlarged autolysosomes, impaired lysosomal protein turnover and reduced cathepsin activity levels [177].

The GG analysis indicated elevated levels of many GGs, GM1, GD1a, and GD1b including the minor gangliosides, GM2 and GM3 [178].

## 6. Drug-Induced GG Accumulation

A long lasting treatment of patients or animals with CADs (cationic amphiphilic drugs) induces a phospholipidosis, a reversible LSD [68]. Only a few studies have investigated the change of the lipid composition and content during drug-induced phospholipidosis. Nilsson et al. demonstrated that phospholipidosis induced by chloroquinone lead to a secondary GG accumulation (10–15-fold) especially of GG GM2 [182].

In reconstitution experiments in vitro, we could show that CADs (desipramine, chlorpromazine, imipramine, and chloroquine) inhibit the catabolism of membrane-bound GM2 by Hex A in the presence of GM2AP [7]. We propose that the increasing accumulation of CAD molecules at the surface of ILVs will reduce their negative surface potential created by anionic phospholipids like BMP even at pH values as low as 5 [10], thereby releasing lysosomal hydrolases and SAPs from the GM2 carrying ILV membrane surfaces [7,38,56] (Figure 1D). A detailed overview about the mechanism of drug induced phospholipidosis is described in [68].

## 7. Conclusions and Perspectives

The simple idea of a monogenetic LSD, a gene defect causes a dysfunctional protein, usually a defective hydrolase, which leads to a progressive accumulation of its undegraded substrate, seems to be rather naïve. The reality is more complex and better described by a cascading model of errors. First of all, most lysosomal proteins, e.g., hydrolases, transfer proteins, and lipid binding proteins (the SAPs) are rather promiscuous, having a rather broad, sometimes overlapping substrate specificity and therefore act on many and not only on few metabolites or even on one only.

Secondly, the sphingolipid cleaving activity of lysosomal hydrolases is effectively regulated by genetic and posttranslational modifiers [6,8] which, however, hardly affect their activity against soluble and synthetic substrates, e.g., MUF derivatives, which are commonly used in vitro to assay patients’ lysosomal hydrolases. At the genetic level, protein cofactors, e.g., the lipid binding SAPs, and at the posttranslational level many factors in the microenvironment of the lysosome crucially affect the sphingolipid cleaving activity of the lysosomal hydrolases. Especially membrane lipids of the ILVs and the electrostatic attraction of cationic, protonated hydrolases, and other needed proteins to the anionic surface of the sphingolipid-substrate carrying ILVs are of the utmost importance. [8].

Thirdly, among the crucial modifiers of sphingolipid catabolic pathways are the primary storage compounds which can trigger further pathological cascading errors in LSDs. The secondary accumulation of metabolites in LSDs, e.g., little GGs, is known for a long time, however, molecular mechanisms have been investigated only recently. So far, inhibitory effects of primary storage material on otherwise intact lysosomal pathways have been observed as the main basis for the secondary accumulation of metabolites. For example, primary and progressively accumulating sphingomyelin triggers an impressive and pathological secondary cholesterol storage in Niemann–Pick disease type A and type B (10). Or a secondary GG GM2 accumulation in the brain is triggered by chondroitin sulfate, a primary storage material in some MPS (Hurler, Hunter, Sanfilippo, Sly syndrome), which is effectively inhibiting the lysosomal GM2 catabolism [7]. It is expected that secondarily accumulating metabolites, depending on their structure and concentration, can also lead to further pathologies in lysosomal functions. These and many other factors can disturb lysosomal metabolism and are open for future research. Just to mention a few: disturbed secretion of metabolic products from the lysosomal system (sugars, fatty acids, amino acids, toxic sphingoid bases like sphingosine, and lysosphingolipids like glucosylsphingosine, etc.), the level of the lysosomal pH-value and its mostly unknown regulation, involvement of intracellular trafficking of proteins, lipids and amino acids, the biogenesis, maturation, and turnover of endosomes and lysosomes, processes of autophagy, the functions of endocytotic and phagocytotic pathways and many more.

Finally, a word on some therapeutic approaches currently under development and further improvement. Enzyme replacement therapy (ERT), has been established for some LSDs not involving the central nervous system. Others like substrate reduction therapy (SRT) and chaperone therapy reduce the increase of storage material, but none of them keeps the promise of a cure. Gene replacement therapy has been successfully studied in animal models and is now slowly being applied to patients. It carries the promise of a cure, but still needs a long way to really cure neurodegenerative diseases like the gangliosidoses.

## Figures and Tables

**Table 1:** Gangliosidoses.

Disease	Protein	Gene	Storage Compound	References
**GM2 Gangliosidoses**				
Tay–Sachs disease (B variant)	Hex A^1^, Hex S^2^	*HEXA*	GM2, SM2a, lyso-GM2, GA2	[28]
B1 variant	Hex A^1^	*HEXA*	GM2	[28]
Sandhoff disease	Hex A^1^, Hex B^3^	*HEXB*	GM2, globoside, oligosaccharides, lyso-GM2	[28,29,30]
GM2AP deficiency (AB variant)	GM2AP	*GM2A*	GM2	[28]
**GM1 Gangliosidosis**	acid β-galactosidase	*GBL1*	GM1, GA1, GM2, GM3, GA1a, lyso GM1 GlcCer Laccer, oligosaccharides, keratan sulfate	[31,32]

^1^ Hex A αβ-subunit, ^2^ Hex S αα-subunit, and ^3^ Hex B ββ-subunit.

**Table 2:** Secondary Ganglioside Accumulation in Patients with LSDs.

Disease	Protein	Gene	Major Storage Compound ^1^	Accumulated Ganglioside	References
**Sphingolipidoses**					
Niemann–Pick disease type A, B	ASM	*SMPD1*	SM^1^	GM2, GM3	[3,32,90]
Niemann–Pick disease type C	NPC1	*NPC1*	Chol ^2^	GM2, GM3, GM1	[4,32,179]
	NPC2	*NPC2*	Chol ^2^	GM2, GM3	[4,95,179]
Gaucher disease	β-glucosidase	*GBA1*	GlcCer ^3^	GM2, GM3, GM1, GD3	[112,180]
Metachromatic leukodystrophy	Arylsulfatase A	*ARSA*	Sulfatide	GM2	[120]
Krabbe disease	galactocerebrosidase	*GALC*	GalCer ^4^	GD2, GD3, GM3	[115]
Farber disease	acid ceramidase	*ASAH1*	ceramide	Gangliosides	[123,124]
**Mucopolysaccharidoses (MPS)**					
MPS Ι (Hurler syndrome)	α-L iduronidase	*IDUA*	heparan sulfate, dermatan sulfate	GM2, GM3	[32,128,133,142]
MPS ΙΙ (Hunter syndrome)	iduronate-2-sulfatase	*IDS*	heparan sulfate, dermatan sulfate	GM2, GM3	[133]
MPS ΙΙΙA (Sanfilippo syndrome)	Heparin-*N*-sulfatase	*SGSH*	heparan sulfate	GM2, GM3, GD2	[133,142]
MPS ΙΙΙB (Sanfilippo syndrome)	α-*N*-Acetylglucosaminidase	*NAGLU*	heparan sulfate	GM2, GM3, GD2	[128]
MPS ΙΙΙC (Sanfilippo syndrome)	Acetyl-CoA: α-*N*-glucosaminide *N*-acetyltransferase	*HGSNAT*	heparan sulfate	GM2, GM3, GD2	
MPS ΙΙΙD (Sanfilippo syndrome)	*N*-Acetylglucosamine-6-sulfatase	*GNS*	heparan sulfate	GM3, GM2, GD2	[136]
MPS VΙ (Maroteaux–Lamy syndrome)	arylsulfatase B	*ASRB*	dermatan sulfate	GM2, GM3	[140]
MPS VΙΙ (Sly syndrome)	β-glucuronidase	*GUSB*	heparan sulfate, dermatan sulfate, chondroitin sulfate	GM2, GM3	[5,142]
**Mucolipidoses**					
Mucolipidosis ΙΙ (I-cell disease) Mucolipidosis ΙΙΙ (pseudo-Hurler polydystrophy)	*N*-acetylglucosamine-1-phosphotransferase	*GNPTAB*		GM1	[143,181]
Mucolipidosis ΙV (mucolipidin 1 deficiency)	TRPML1	*MCOLN1*		GM3, GD1a	[147]
**Glycoproteinoses**					
Galactosialidosis	lysosomal protective protein–cathepsin A (PPCA)	*CTSA*	sialyloligosacchaides	GM2, GM3, GM1, GD1a	[149]
α-Mannosidosis	α-D-mannosidase	*MAN2B1*	mannose-rich oligosaccharides	GM2, GM3	[32,152]
Sialidosis	acid neuraminidase 1	*NEU1*	sialyloligosaccharides, sialoglycoproteins	GM3, GD3, GM4, LM1	[151]
**Neuronal ceroid lipofuscinoses (NCL)**					
NCL 3 (Batten disease)	CLN3	*CLN3*	ATPase subunit c, lipofuscin	GM3	[159]
NCL 6	CLN 6	*NCLF*	ATPase subunit c, lipofuscin	GM2, GM3	[156]
NCL 10 (Congenital cathepsin D deficiency)	Cathapsin D	*CTSD*	ATPase subunit c, Sap A, Sap D, lipofuscin	GM2, GM3	[156]
**Hereditary spastic paraplegia (HSP)**					
HSP type SPG 11	spatacsin	*SPG11*	p62	GM2, GM3, GD2, GD3	[171]
**Alzheimer**					
TgCRND8 (Alzheimer maus)			Aβ40, Aβ42	GM1, GD1a, GD1b, GM2, GM3	[178]

^1^ SM, sphingomyelin; ^2^ Chol, cholesterol; ^3^ GlcCer, glycosylceramide; ^4^ GalCer, galactosylceramide.

## Abbreviations

ASM	Acid sphingomyelinase
BMP	Bis(monoacylglycero)phosphate
CAD	Cationic amphiphilic drug
GBA1	Glucosylceramide-β-glucosidase
GG	Ganglioside
GSL	Glycosphingolipid
GM2AP	GM2 activator protein
Hex A	β-Hexosaminidase A
Hex B	β-Hexosaminidase B
HSP	Hereditary spastic paraplegia
IVL	Intralysosomal luminal vesicle
LSD	Lysosomal storage disorders
MLD	Metachromatic leukodystrophy
MPS	Mucopolysaccharidoses
NCL	Neuronal ceroid lipofuscinoses
NEU	Neuraminidase
NPC1	Niemann–Pick disease protein C type 1
NPC2	Niemann–Pick disease protein C type 2
SAP	Sphingolipid activator protein
Sap	Saposin
SD	Sandhoff disease
TSD	Tay–Sachs disease

## References

[1] Lehovský M. (1972). Clinical pictures in patients with thesaurismosis of gangliosides. Rev. Czech. Med..

[2] Van Bogaert L., Klenk E. (1953). Clinical, histopathological and chemical aspects of phosphatide thesaurismosis. G Psichiatr. Neuropatol..

[3] Schuchman E.H., Desnick R.J., Scriver C.R., Beaudet A.L., Sly W.S., Valle D. (2001). Niemann–Pick disease types A and B: Sphingomyelinase deficiencies. The Metabolic and Molecular Bases of Inherited Disease.

[4] Patterson M.C., Vanier M.T., Suzuki K., Morris J.A., Eugene C., Neufeld E.B., Blanchette-Mackie Joan E., Pentchev P.G., Scriver C.R., Beaudet A.L., Sly W.S., Valle D. (2001). Niemann–Pick disease type C: A lipid trafficking disorders. The Metabolic and Molecular Bases of Inherited Disease.

[5] Neufeld E., Muenzer J., Scriver C.R., Beaudet A.L., Sly W.S., Valle D. (2001). The mucopolysaccharidoses. The Metabolic and Molecular Bases of Inherited Disease.

[6] Anheuser S., Breiden B., Sandhoff K. (2019). Membrane lipids and their degradation compounds control GM2 catabolism at intralysosomal luminal vesicles. J. Lipid Res..

[7] Anheuser S., Breiden B., Sandhoff K. (2019). Ganglioside GM2 catabolism is inhibited by storage compounds of mucopolysaccharidoses and by cationic amphiphilic drugs. Mol. Genet. Metab.

[8] Breiden B., Sandhoff K. (2019). Lysosomal glycosphingolipid storage diseases. Annu. Rev. Biochem..

[9] Sandhoff R., Sandhoff K. (2018). Emerging concepts of ganglioside metabolism. FEBS Lett..

[10] Oninla V.O., Breiden B., Babalola J.O., Sandhoff K. (2014). Acid sphingomyelinase activity is regulated by membrane lipids and facilitates cholesterol transfer by NPC2. J. Lipid Res..

[11] Sachs B. (1887). On arrested cerebral development with speciel reference to its cortical pathology. J. Neur. Ment. Dis..

[12] Sachs B. (1896). A family form of idiocy, generally fatal, associated with early blindness (amaurotic family idiocy). J. Nerv. Ment. Dis..

[13] Klenk E. (1939). Niemann–Pick’sche Krankheit und Amaurotische Idiotie. Hoppe-Seyler’s Z Physiol. Chem..

[14] Sandhoff K., Harzer K. (2013). Gangliosides and gangliosidoses: Principles of molecular and metabolic pathogenesis. J. Neurosci..

[15] Tay W. (1881). Symmetrical changes in the region of the yellow spot in each eye of an infant. Trans. Ophthalmol. Soc..

[16] Jatzkewitz H., Sandhoff K. (1963). On a biochemically special form of infantile amaturotic idiocy. Biochim. Biophys. Acta..

[17] Sandhoff K. (1965). Die Amaurotische Idiotie des Menschen als Störung im Glykosphingolipidstoffwechsel. Doctoral Thesis.

[18] Kuhn R., Wiegandt H. (1963). Die Konstitution der Ganglio-N-tetraose und des Gangliosids GI. Chem. Ber..

[19] Sandhoff K. (2001). The GM2-gangliosidoses and the elucidation of the beta-hexosaminidase system. Adv. Genet..

[20] Sandhoff K. (1969). Variation of beta-N-acetylhexosaminidase-pattern in Tay–Sachs disease. FEBS Lett..

[21] Sandhoff K., Harzer K., Wässle W., Jatzkewitz H. (1971). Enzyme alterations and lipid storage in three variants of Tay–Sachs disease. J. Neurochem..

[22] Conzelmann E., Sandhoff K. (1978). AB variant of infantile GM2 gangliosidosis: Deficiency of a factor necessary for stimulation of hexosaminidase A-catalyzed degradation of ganglioside GM2 and glycolipid GA2. Proc. Natl. Acad. Sci. USA.

[23] Okada S., O’Brien J.S. (1969). Tay–Sachs disease: Generalized absence of a beta-D-N-acetylhexosaminidase component. Science.

[24] Sandhoff K. (1970). The hydrolysis of Tay–Sachs ganglioside (TSG) by human N-acetyl-beta-D-hexosaminidase A. FEBS Lett..

[25] Sandhoff R., Schulze H., Sandhoff K. (2018). Ganglioside metabolism in health and disease. Prog. Mol. Biol. Transl. Sci..

[26] Sandhoff K., Jatzkewitz H., Peters G. (1969). Die infantile amaurotische Idiotie und verwandte Formen als Gangliosid-Speicherkrankheiten. Naturwissenschaften.

[27] Hepbildikler S.T., Sandhoff R., Kölzer M., Proia R.L., Sandhoff K. (2002). Physiological substrates for human lysosomal beta -hexosaminidase S. J. Biol. Chem..

[28] Gravel R., Kaback M.M., Proia R.L., Sandhoff K., Suzuki K., Suzuki K., Scriver C.R., Beaudet A.L., Sly W.S., Valle D. (2001). The GM2 gangliosidoses. The Metabolic and Molecular Bases of Inherited Disease.

[29] Sandhoff K., Andreae U., Jatzkewitz H. (1968). Deficient hexosaminidase activity in an exceptional case of Tay–Sachs disease with additional storage of kidney globoside in visceral organs. Pathol Eur..

[30] Pilz H., Müller D., Sandhoff K., ter Meulen V. (1968). Tay–Sachssche Krankheit mit Hexosaminidase-Defekt. Klinische, morphologische und biochemische Befunde bei einem Fall mit viszeraler Speicherung von Nierenglobosid. Dtsch Med. Wochenschr.

[31] Suzuki K., Chen G.C. (1968). GM1-gangliosidosis (generalized gangliosidosis). Morphology and chemical pathology. Pathol Eur..

[32] Siegel D.A., Walkley S.U. (1994). Growth of ectopic dendrites on cortical pyramidal neurons in neuronal storage diseases correlates with abnormal accumulation of GM2 ganglioside. J. Neurochem..

[33] Kytzia H.J., Hinrichs U., Maire I., Suzuki K., Sandhoff K. (1983). Variant of GM2-gangliosidosis with hexosaminidase A having a severely changed substrate specificity. Embo J..

[34] Kytzia H.J., Sandhoff K. (1985). Evidence for two different active sites on human beta-hexosaminidase A. Interaction of GM2 activator protein with beta-hexosaminidase A. J. Biol. Chem..

[35] Sango K., Yamanaka S., Hoffmann A., Okuda Y., Grinberg A., Westphal H., McDonald M.P., Crawley J.N., Sandhoff K., Suzuki K. (1995). Mouse models of Tay–Sachs and Sandhoff diseases differ in neurologic phenotype and ganglioside metabolism. Nat. Genet..

[36] Jeyakumar M., Smith D., Eliott-Smith E., Cortina-Borja M., Reinkensmeier G., Butters T.D., Lemm T., Sandhoff K., Perry V.H., Dwek R.A. (2002). An inducible mouse model of late onset Tay–Sachs disease. Neurobiol. Dis..

[37] Sango K., McDonald M.P., Crawley J.N., Mack M.L., Tifft C.J., Skop E., Starr C.M., Hoffmann A., Sandhoff K., Suzuki K. (1996). Mice lacking both subunits of lysosomal [beta]-hexosaminidase display gangliosidosis and mucopolysaccharidosis. Nat. Genet..

[38] Anheuser S., Breiden B., Schwarzmann G., Sandhoff K. (2015). Membrane lipids regulate ganglioside GM2 catabolism and GM2 activator protein activity. J. Lipid Res..

[39] O’Brien J.S., Stern M.B., Landing B.H., O’Brien J.K., Donnell G.N. (1965). Generalized gangliosidosis: Another inborn error of ganglioside metabolism?. Am. J. Dis. Child..

[40] Lawrence R., Van Vleet J.L., Mangini L., Harris A., Martin N., Clark W., Chandriani S., LeBowitz J.H., Giugliani R., d’Azzo A. (2019). Characterization of glycan substrates accumulating in GM1 gangliosidosis. Mol. Genet. Metab. Rep..

[41] Wilkening G., Linke T., Uhlhorn-Dierks G., Sandhoff K. (2000). Degradation of membrane-bound ganglioside GM1. Stimulation by bis(monoacylglycero)phosphate and the activator proteins SAP-B and GM2-AP. J. Biol. Chem..

[42] Miyagi T., Takahashi K., Yamamoto K., Shiozaki K., Yamaguchi K. (2018). Biological and pathological roles of ganglioside sialidases. Prog. Mol. Biol. Transl. Sci..

[43] Timur Z.K., Akyildiz Demir S., Marsching C., Sandhoff R., Seyrantepe V. (2015). Neuraminidase-1 contributes significantly to the degradation of neuronal B-series gangliosides but not to the bypass of the catabolic block in Tay–Sachs mouse models. Mol. Genet. Metab. Rep..

[44] Smutova V., Albohy A., Pan X., Korchagina E., Miyagi T., Bovin N., Cairo C.W., Pshezhetsky A.V. (2014). Structural basis for substrate specificity of mammalian neuraminidases. PLoS ONE.

[45] Monti E., Bonten E., D’Azzo A., Bresciani R., Venerando B., Borsani G., Schauer R., Tettamanti G. (2010). Sialidases in vertebrates: A family of enzymes tailored for several cell functions. Adv. Carbohydr. Chem. Biochem..

[46] D’Azzo A., Machado E., Annunziata I. (2015). Pathogenesis, emerging therapeutic targets and treatment in sialidosis. Expert Opin. Orphan. Drugs..

[47] Bonten E., van der Spoel A., Fornerod M., Grosveld G., d’Azzo A. (1996). Characterization of human lysosomal neuraminidase defines the molecular basis of the metabolic storage disorder sialidosis. Genes Dev..

[48] D’Azzo A., Bonten E. (2010). Molecular mechanisms of pathogenesis in a glycosphingolipid and a glycoprotein storage disease. Biochem Soc. Trans..

[49] Fingerhut R., van der Horst G.T.J., Verheijen F.W., Conzelmann E. (1992). Degradation of gangliosides by the lysosomal sialidase requires an activator protein. Eur J. Biochem..

[50] Abdul-Hammed M., Breiden B., Schwarzmann G., Sandhoff K. (2017). Lipids regulate the hydrolysis of membrane bound glucosylceramide by lysosomal β-glucocerebrosidase. J. Lipid Res..

[51] Akiyama H., Kobayashi S., Hirabayashi Y., Murakami-Murofushi K. (2013). Cholesterol glucosylation is catalyzed by transglucosylation reaction of ß-glucosidase 1. Biochem. Biophys. Res. Com..

[52] Eskelinen E.-L., Tanaka Y., Saftig P. (2003). At the acidic edge: Emerging functions for lysosomal membrane proteins. Trends Cell. Biol..

[53] Kobayashi T., Beuchat M.H., Lindsay M., Frias S., Palmiter R.D., Sakuraba H., Parton R.G., Gruenberg J. (1999). Late endosomal membranes rich in lysobisphosphatidic acid regulate cholesterol transport. Nat. Cell Biol..

[54] Möbius W., van Donselaar E., Ohno-Iwashita Y., Shimada Y., Heijnen H.F., Slot J.W., Geuze H.J. (2003). Recycling compartments and the internal vesicles of multivesicular bodies harbor most of the cholesterol found in the endocytic pathway. Traffic (Copenhagen, Denmark).

[55] Graf C.G.F., Schulz C., Schmälzlein M., Heinlein C., Mönnich M., Perkams L., Püttner M., Boos I., Hessefort M., Lombana Sanchez J.N. (2017). Synthetic glycoforms reveal carbohydrate-dependent bioactivity of human saposin D. Angew Chem. Int. Ed. Engl..

[56] Kölzer M., Werth N., Sandhoff K. (2004). Interactions of acid sphingomyelinase and lipid bilayers in the presence of the tricyclic antidepressant desipramine. FEBS Lett..

[57] Yu C., Sun Q., Zhou H. (2013). Enzymatic screening and diagnosis of lysosomal storage diseases. N. Am. J. Med. Sci...

[58] Galjaard H. (1980). Genetic metabolic diseases: Early diagnosis and prenatal analysis.

[59] Kolter T., Proia R.L., Sandhoff K. (2002). Combinatorial ganglioside biosynthesis. J. Biol. Chem..

[60] Van Meer G., Voelker D.R., Feigenson G.W. (2008). Membrane lipids: Where they are and how they behave. Nat. Rev. Mol. Cell Biol..

[61] Abdul-Hammed M., Breiden B., Adebayo M.A., Babalola J.O., Schwarzmann G., Sandhoff K. (2010). Role of endosomal membrane lipids and NPC2 in cholesterol transfer and membrane fusion. J. Lipid Res..

[62] Wang M.L., Motamed M., Infante R.E., Abi-Mosleh L., Kwon H.J., Brown M.S., Goldstein J.L. (2010). Identification of surface residues on Niemann–Pick C2 essential for hydrophobic handoff of cholesterol to NPC1 in lysosomes. Cell Metab..

[63] Vanier M.T. (1983). Biochemical studies in Niemann–Pick disease I. Major sphingolipids of liver and spleen. Biochim. Biophys Acta..

[64] Jatzkewitz H., Pilz H., Sandhoff K. (1965). Quantitative Bestimmungen von Gangliosiden und ihren Neuraminsäurefreien Derivaten bei infantilen, juvenilen und adulten Formen der amaurotischen Idiotie und einer spätinfantilen biochemischen Sonderform. J. Neurochem..

[65] Remmel N., Locatelli-Hoops S., Breiden B., Schwarzmann G., Sandhoff K. (2007). Saposin B mobilizes lipids from cholesterol-poor and bis(monoacylglycero)phosphate-rich membranes at acidic pH. Unglycosylated patient variant saposin B lacks lipid-extraction capacity. FEBS J..

[66] Locatelli-Hoops S., Remmel N., Klingenstein R., Breiden B., Rossocha M., Schoeniger M., Koenigs C., Saenger W., Sandhoff K. (2006). Saposin A mobilizes lipids from low cholesterol and high bis(monoacylglycerol)phosphate-containing membranes: Patient variant Saposin A lacks lipid extraction capacity. J. Biol. Chem..

[67] Walkley S.U. (2009). Pathogenic cascades in lysosomal disease-Why so complex?. J. Inherit. Metab. Dis..

[68] Breiden B., Sandhoff K. (2020). Emerging mechanisms of drug-induced phospholipidosis. Biol. Chem..

[69] Sarmientos F., Schwarzmann G., Sandhoff K. (1986). Specificity of human glucosylceramide beta-glucosidase towards synthetic glucosylsphingolipids inserted into liposomes. Kinetic studies in a detergent-free assay system. Eur J. Biochem..

[70] Walkley S.U., Vanier M.T. (2009). Secondary lipid accumulation in lysosomal disease. Biochim. Biophys. Acta..

[71] Tessitore A., del P.M.M., Sano R., Ma Y., Mann L., Ingrassia A., Laywell E.D., Steindler D.A., Hendershot L.M., d’Azzo A. (2004). GM1-ganglioside-mediated activation of the unfolded protein response causes neuronal death in a neurodegenerative gangliosidosis. Mol. Cell..

[72] Jmoudiak M., Futerman A.H. (2005). Gaucher disease: Pathological mechanisms and modern management. Br. J. Haematol..

[73] Schulze H., Sandhoff K. (2011). Lysosomal lipid storage diseases. Cold Spring Harb Perspect Biol.

[74] Toulmay A., Prinz W.A. (2011). Lipid transfer and signaling at organelle contact sites: The tip of the iceberg. Curr. Opin. Cell Biol..

[75] Hanada K. (2020). Organelle contacts: Sub-organelle zones to facilitate rapid and accurate inter-organelle trafficking of lipids. Traffic..

[76] Liu E.A., Lieberman A.P. (2019). The intersection of lysosomal and endoplasmic reticulum calcium with autophagy defects in lysosomal diseases. Neurosci. Lett..

[77] Ballabio A., Gieselmann V. (2009). Lysosomal disorders: From storage to cellular damage. Biochim. Biophys. Acta..

[78] Pelled D., Riebeling C., van Echten-Deckert G., Sandhoff K., Futerman A.H. (2003). Reduced rates of axonal and dendritic growth in embryonic hippocampal neurones cultured from a mouse model of Sandhoff disease. Neuropathol. Appl. Neurobiol..

[79] Virgolini M.J., Feliziani C., Cambiasso M.J., Lopez P.H., Bollo M. (2019). Neurite atrophy and apoptosis mediated by PERK signaling after accumulation of GM2-ganglioside. Biochim. Biophys. Acta..

[80] Takamura A., Higaki K., Kajimaki K., Otsuka S., Ninomiya H., Matsuda J., Ohno K., Suzuki Y., Nanba E. (2008). Enhanced autophagy and mitochondrial aberrations in murine G(M1)-gangliosidosis. Biochem. Biophys. Res. Commun..

[81] Niemann A. (1914). Un unbekanntes Krankheitsbild. Jahrb. Kinderheilkd..

[82] Klenk E. (1934). Über die Natur der Phosphatide der Milz bei der Niemann–Pickschen Krankheit. Hoppe Seylers Z Physiol. Chem..

[83] Brady R.O., Kanfer J., Mock M., Fredrickson D. (1966). The metabolism of sphingomyelin. Evidence of an enzymatic deficiency in Niemann–Pick disease. Proc. Natl. Acad. Sci. USA.

[84] Quintern L.E., Weitz G., Nehrkorn H., Tager J.M., Schram A.W., Sandhoff K. (1987). Acid sphingomyelinase from human urine: Purification and characterization. Biochim. Biophys. Acta..

[85] Ferlinz K., Hurwitz R., Moczall H., Lansmann S., Schuchman E.H., Sandhoff K. (1997). Functional characterization of the N-glycosylation sites of human acid sphingomyelinase by site-directed mutagenesis. Eur. J. Biochem..

[86] Hurwitz R., Ferlinz K., Vielhaber G., Moczall H., Sandhoff K. (1994). Processing of human acid sphingomyelinase in normal and I-cell fibroblasts. J. Biol. Chem..

[87] Quintern L.E., Schuchman E.H., Levran O., Suchi M., Ferlinz K., Reinke H., Sandhoff K., Desnick R.J. (1989). Isolation of cDNA clones encoding human acid sphingomyelinase: Occurrence of alternatively processed transcripts. Embo J..

[88] Ferlinz K., Hurwitz R., Sandhoff K. (1991). Molecular basis of acid sphingomyelinase deficiency in a patient with Niemann–Pick disease type A. Biochem. Biophys. Res. Commun..

[89] Levran O., Desnick R., Schuchman E. (1991). Niemann–Pick disease: A frequent missense mutation in the acid sphingomyelinase gene of Ashkenazi Jewish type A and B patiens. Proc. Natl. Acad. Sci. USA.

[90] Greenbaum M., Hoffman L.M., Schneck L. (1976). Ceramide hexosides in Niemann–Pick disease brain. J. Neurol..

[91] Vellodi A., Hobbs J.R., O’Donnell N.M., Coulter B.S., Hugh-Jones K. (1987). Treatment of Niemann–Pick disease type B by allogeneic bone marrow transplantation. Br. Med. J..

[92] Schuchman E.H., Desnick R.J. (2017). Types A and B Niemann–Pick disease. Mol. Genet. Metab..

[93] Samaranch L., Perez-Canamas A., Soto-Huelin B., Sudhakar V., Jurado-Arjona J., Hadaczek P., Avila J., Bringas J.R., Casas J., Chen H. (2019). Adeno-associated viral vector serotype 9-based gene therapy for Niemann–Pick disease type A. Sci. Transl. Med..

[94] Vanier M.T. (2015). Complex lipid trafficking in Niemann–Pick disease type C. J. Inherit. Metab. Dis..

[95] Sleat D., Wiseman J., El Banna M., Price S., Verot L., Shen M., Tint G., Vanier M., Walkley S., Lobel P. (2004). Genetic evidence for nonredundant functional cooperativity between NPC1 and NPC2 in lipid transport. Proc. Natl Acad Sci USA.

[96] Carstea E.D., Morris J.A., Coleman K.G., Loftus S.K., Zhang D., Cummings C., Gu J., Rosenfeld M.A., Pavan W.J., Krizman D.B. (1997). Niemann–Pick C1 disease gene: Homology to mediators of cholesterol homeostasis. Science.

[97] Naureckiene S., Sleat D.E., Lackland H., Fensom A., Vanier M.T., Wattiaux R., Jadot M., Lobel P. (2000). Identification of HE1 as the second gene of Niemann–Pick C disease. Science.

[98] Lloyd-Evans E., Morgan A.J., He X., Smith D.A., Elliot-Smith E., Sillence D.J., Churchill G.C., Schuchman E.H., Galione A., Platt F.M. (2008). Niemann–Pick disease type C1 is a sphingosine storage disease that causes deregulation of lysosomal calcium. Nat. Med..

[99] Zschoche A., Fürst W., Schwarzmann G., Sandhoff K. (1994). Hydrolysis of lactosylceramide by human galactosylceramidase and GM1-beta-galactosidase in a detergent-free system and its stimulation by sphingolipid activator proteins, sap-B and sap-C. Activator proteins stimulate lactosylceramide hydrolysis. Eur. J. Biochem..

[100] Hurwitz R., Ferlinz K., Sandhoff K. (1994). The tricyclic antidepressant desipramine causes proteolytic degradation of lysosomal sphingomyelinase in human fibroblasts. Biol. Chem. Hoppe Seyler.

[101] Bhuvaneswaran C., Venkatesan S., Mitropoulos K.A. (1985). Lysosomal accumulation of cholesterol and sphingomyelin: Evidence for inhibition of acid sphingomyelinase. Eur. J. Cell Biol..

[102] Shen D., Wang X., Li X., Zhang X., Yao Z., Dibble S., Dong X.P., Yu T., Lieberman A.P., Showalter H.D. (2012). Lipid storage disorders block lysosomal trafficking by inhibiting a TRP channel and lysosomal calcium release. Nat. Commun..

[103] Sitarska D., Ługowska A. (2019). Laboratory diagnosis of the Niemann–Pick type C disease: An inherited neurodegenerative disorder of cholesterol metabolism. Metab. Brain Dis..

[104] Deodato F., Boenzi S., Taurisano R., Semeraro M., Sacchetti E., Carrozzo R., Dionisi-Vici C. (2018). The impact of biomarkers analysis in the diagnosis of Niemann–Pick C disease and acid sphingomyelinase deficiency. Clinica. Chimica. Acta..

[105] Hammerschmidt T.G., de Oliveira Schmitt Ribas G., Saraiva-Pereira M.L., Bonatto M.P., Kessler R.G., Souza F.T.S., Trapp F., Michelin-Tirelli K., Burin M.G., Giugliani R. (2018). Molecular and biochemical biomarkers for diagnosis and therapy monitorization of Niemann–Pick type C patients. Int. J. Dev. Neurosci. Off. J. Int. Soc. Dev. Neurosci..

[106] Nilsson O., Svennerholm L. (1982). Accumulation of glucosylceramide and glucosylsphingosine (Psychosine) in gerebrum and gerebellum in infantile and juvenile Gaucher disease. J. Neurochem..

[107] Brady R.O., Kanfer J.N., Shapiro D. (1965). Metabolism of glucocerebrosides. Evidence of an enzymatic deficiency in Gaucher’s disease. Biochem. Biophys. Res. Com..

[108] Barton N.W., Brady R.O., Dambrosia J.M., Di Bisceglie A.M., Doppelt S.H., Hill S.C., Mankin H.J., Murray G.J., Parker R.I., Argoff C.E. (1991). Replacement therapy for inherited enzyme deficiency — Macrophage-targeted glucocerebrosidase for Gaucher’s disease. N Engl. J. Med..

[109] Doering T., Proia R.L., Sandhoff K. (1999). Accumulation of protein-bound epidermal glucosylceramides in beta-glucocerebrosidase deficient type 2 Gaucher mice. FEBS Lett..

[110] Breiden B., Sandhoff K. (2014). The role of sphingolipid metabolism in cutaneous permeability barrier formation. Biochim. Biophys. Acta..

[111] Ghauharali-van der Vlugt K., Langeveld M., Poppema A., Kuiper S., Hollak C.E., Aerts J.M., Groener J.E. (2008). Prominent increase in plasma ganglioside GM3 is associated with clinical manifestations of type I Gaucher disease. Clin. Chim. Acta..

[112] Gornati R., Berra B., Montorfano G., Martini C., Ciana G., Ferrari P., Romano M., Bembi B. (2002). Glycolipid analysis of different tissues and cerebrospinal fluid in type II Gaucher disease. J. Inherit. Metab. Dis..

[113] Rolfs A., Giese A.K., Grittner U., Mascher D., Elstein D., Zimran A., Bottcher T., Lukas J., Hubner R., Golnitz U. (2013). Glucosylsphingosine is a highly sensitive and specific biomarker for primary diagnostic and follow-up monitoring in Gaucher disease in a non-Jewish, Caucasian cohort of Gaucher disease patients. PLoS ONE.

[114] Hurvitz N., Dinur T., Becker-Cohen M., Cozma C., Hovakimyan M., Oppermann S., Demuth L., Rolfs A., Abramov A., Zimran A. (2019). Glucosylsphingosine (lyso-Gb1) as a Biomarker for Monitoring Treated and Untreated Children with Gaucher Disease. Int. J. Mol. Sci..

[115] Vanier M.T., Svennerholm L. (1975). Chemical pathology of Krabbe’s disease. Ceramide-hexosides and gangliosides of brain. Acta Paediatr Scand..

[116] Escolar M.L., Kiely B.T., Shawgo E., Hong X., Gelb M.H., Orsini J.J., Matern D., Poe M.D. (2017). Psychosine, a marker of Krabbe phenotype and treatment effect. Mol. Genet. Metab..

[117] Mikulka C.R., Sands M.S. (2016). Treatment for Krabbe’s disease: Finding the combination. J. Neurosci. Res..

[118] Von Figura K., Gieselmann V., Jaeken J., Scriver C.R., Beaudet A.L., Sly W.S., Valle D. (2001). Metachroimatic leukodysrtophy. The Metabolic and Molecular Bases of Inherited Disease.

[119] Suzuki K. (1967). Ganglioside patterns of normal and pathological brains. Inborn Disorders of Sphingolipid Metabolism.

[120] Sevin C., Verot L., Benraiss A., Van Dam D., Bonnin D., Nagels G., Fouquet F., Gieselmann V., Vanier M.T., De Deyn P.P. (2007). Partial cure of established disease in an animal model of metachromatic leukodystrophy after intracerebral adeno-associated virus-mediated gene transfer. Gene Therapy.

[121] Beerepoot S., Nierkens S., Boelens J.J., Lindemans C., Bugiani M., Wolf N.I. (2019). Peripheral neuropathy in metachromatic leukodystrophy: Current status and future perspective. Orphanet. J. Rare Dis..

[122] Klein A., Henseler M., Klein C., Suzuki K., Harzer K., Sandhoff K. (1994). Sphingolipid activator protein D (sap-D) stimulates the lysosomal degradation of ceramide in vivo. Biochem. Biophys. Res. Commun..

[123] Ferreira C.R., Gahl W.A. (2017). Lysosomal storage diseases. Transl. Sci. Rare Dis..

[124] Antonarakis S.E., Valle D., Moser H.W., Moser A., Qualman S.J., Zinkham W.H. (1984). Phenotypic variability in siblings with Farber disease. J. Pediatr..

[125] Cozma C., Iurașcu M.-I., Eichler S., Hovakimyan M., Brandau O., Zielke S., Böttcher T., Giese A.-K., Lukas J., Rolfs A. (2017). C26-Ceramide as highly sensitive biomarker for the diagnosis of Farber Disease. Sci. Rep..

[126] He X., Dworski S., Zhu C., DeAngelis V., Solyom A., Medin J.A., Simonaro C.M., Schuchman E.H. (2017). Enzyme replacement therapy for Farber disease: Proof-of-concept studies in cells and mice. BBA Clin..

[127] Fujita N., Suzuki K., Vanier M.T., Popko B., Maeda N., Klein A., Henseler M., Sandhoff K., Nakayasu H. (1996). Targeted disruption of the mouse sphingolipid activator protein gene: A complex phenotype, including severe leukodystrophy and wide-spread storage of multiple sphingolipids. Hum. Mol. Genet..

[128] Constantopoulos G., Dekaban A.S. (1978). Neurochemistry of the mucopolysaccharidoses: Brain lipids and lysosomal enzymes in patients with four types of mucopolysaccharidosis and in normal controls. J. Neurochem..

[129] Stapleton M., Arunkumar N., Kubaski F., Mason R.W., Tadao O., Tomatsu S. (2018). Clinical presentation and diagnosis of mucopolysaccharidoses. Mol. Genet. Metab.

[130] Donati M.A., Pasquini E., Spada M., Polo G., Burlina A. (2018). Newborn screening in mucopolysaccharidoses. Ital. J. Pediatrics.

[131] Zhang H., Wood T., Young S.P., Millington D.S. (2015). A straightforward, quantitative ultra-performance liquid chromatography-tandem mass spectrometric method for heparan sulfate, dermatan sulfate and chondroitin sulfate in urine: An improved clinical screening test for the mucopolysaccharidoses. Mol. Genet. Metab..

[132] Kiely B.T., Kohler J.L., Coletti H.Y., Poe M.D., Escolar M.L. (2017). Early disease progression of Hurler syndrome. Orphanet. J. Rare Dis..

[133] Constantopoulos G., Iqbal K., Dekaban A.S. (1980). Mucopolysaccharidosis types IH, IS, II and IIIA: Glycosaminoglycans and lipids of isolated brain cells and other fractions from autopsied tissues. J. Neurochem..

[134] Wraith J.E., Scarpa M., Beck M., Bodamer O.A., De Meirleir L., Guffon N., Meldgaard Lund A., Malm G., Van der Ploeg A.T., Zeman J. (2008). Mucopolysaccharidosis type II (Hunter syndrome): A clinical review and recommendations for treatment in the era of enzyme replacement therapy. Eur. J. Pediatrics.

[135] Saville J.T., Flanigan K.M., Truxal K.V., McBride K.L., Fuller M. (2019). Evaluation of biomarkers for Sanfilippo syndrome. Mol. Genet. Metab..

[136] Liour S.S., Jones M.Z., Suzuki M., Bieberich E., Yu R.K. (2001). Metabolic studies of glycosphingolipid accumulation in mucopolysaccharidosis IIID. Mol. Genet. Metab..

[137] Gaffke L., Pierzynowska K., Piotrowska E., Węgrzyn G. (2018). How close are we to therapies for Sanfilippo disease?. Metab. Brain Dis..

[138] Lieberman A.P., Puertollano R., Raben N., Slaugenhaupt S., Walkley S.U., Ballabio A. (2012). Autophagy in lysosomal storage disorders. Autophagy.

[139] Tessitore A., Pirozzi M., Auricchio A. (2009). Abnormal autophagy, ubiquitination, inflammation and apoptosis are dependent upon lysosomal storage and are useful biomarkers of mucopolysaccharidosis VI. Pathogenetics..

[140] Walkley S.U. (2004). Secondary accumulation of gangliosides in lysosomal storage disorders. Semin Cell Dev. Biol.

[141] Harmatz P., Whitley C.B., Waber L., Pais R., Steiner R., Plecko B., Kaplan P., Simon J., Butensky E., Hopwood J.J. (2004). Enzyme replacement therapy in mucopolysaccharidosis VI (Maroteaux–Lamy syndrome). J. Pediatrics.

[142] McGlynn R., Dobrenis K., Walkley S.U. (2004). Differential subcellular localization of cholesterol, gangliosides, and glycosaminoglycans in murine models of mucopolysaccharide storage disorders. J. Comp. Neurol..

[143] Dacremont G., Kint J.A., Cocquyt G. (1974). Brain sphingolipids in I cell disease (mucolipidosis II). J. Neurochem..

[144] LaPlante J.M., Falardeau J., Sun M., Kanazirska M., Brown E.M., Slaugenhaupt S.A., Vassilev P.M. (2002). Identification and characterization of the single channel function of human mucolipin-1 implicated in mucolipidosis type IV, a disorder affecting the lysosomal pathway. FEBS Lett..

[145] Boudewyn L.C., Walkley S.U. (2019). Current concepts in the neuropathogenesis of mucolipidosis type IV. J. Neurochem..

[146] Cam L., Tettamanti G., Berra B., Sale F.O., Borrone C., Gatti R., Durand P., Martin J.J. (1982). Mucolipidosis IV, a sialolipidosis due to ganglioside sialidase deficiency. J. Inherit. Metab. Dis..

[147] Tellez-Nagel I., Rapin I., Iwamoto T., Johnson A.B., Norton W.T., Nitowsky H. (1976). Mucolipidosis IV. Clinical, ultrastructural, histochemical, and chemical studies of a case, including a brain biopsy. Arch. Neurol..

[148] D’Azzo A., Andria G., Strisciuglio P., Galjaard H., Scriver C., Beaudet A., Sly W., Valle D. (2001). Galactosialidosis. The Metabolic and Molecular Bases of Inherited Disease.

[149] Yoshino H., Miyashita K., Miyatani N., Ariga T., Hashimoto Y., Tsuji S., Oyanagi K., Ohama E., Ikuta F., Suzuki A. (1990). Abnormal glycosphingolipid metabolism in the nervous system of galactosialidosis. J. Neurol. Sci..

[150] Miyatake T., Atsumi T., Obayashi T., Mizuno Y., Ando S., Ariga T., Matsui-Nakamura K., Yamada T. (1979). Adult type neuronal storage disease with neuraminidase deficiency. Ann. Neurol..

[151] Thomas G.H., Scriver C.R., Beaudet A.L., Sly W.S., Valle D. (2001). Disorders of glycoprotein degradation: α-mannosidosis, β-mannosidosis, fucosidosis, and sialidosis. The Metabolic and Molecular Bases of Inherited Disease.

[152] Goodman L.A., Livingston P.O., Walkley S.U. (1991). Ectopic dendrites occur only on cortical pyramidal cells containing elevated GM2 ganglioside in alpha-mannosidosis. Proc. Natl Acad Sci USA.

[153] Harmatz P., Cattaneo F., Ardigò D., Geraci S., Hennermann J.B., Guffon N., Lund A., Hendriksz C.J., Borgwardt L. (2018). Enzyme replacement therapy with velmanase alfa (human recombinant alpha-mannosidase): Novel global treatment response model and outcomes in patients with alpha-mannosidosis. Mol. Genet. Metab..

[154] Ceccarini M.R., Codini M., Conte C., Patria F., Cataldi S., Bertelli M., Albi E., Beccari T. (2018). Alpha-Mannosidosis: Therapeutic Strategies. Int. J. Mol. Sci..

[155] Hofmann S.L., Peltonen L., Scriver C.R., Beaudet A.L., Sly W.S., Valle D. (2001). The neuronal ceroid lipofuscinosis. The Metabolic and Molecular Bases of Inherited Disease.

[156] Jabs S., Quitsch A., Käkelä R., Koch B., Tyynelä J., Brade H., Glatzel M., Walkley S., Saftig P., Vanier M.T. (2008). Accumulation of bis(monoacylglycero)phosphate and gangliosides in mouse models of neuronal ceroid lipofuscinosis. J. Neurochem..

[157] Walkley S.U. (1998). Cellular pathology of lysosomal storage disorders. Brain Pathol..

[158] Kyttälä A., Lahtinen U., Braulke T., Hofmann S.L. (2006). Functional biology of the neuronal ceroid lipofuscinoses (NCL) proteins. Biochim. Biophys. Acta..

[159] Somogyi A., Petcherski A., Beckert B., Huebecker M., Priestman D.A., Banning A., Cotman S.L., Platt F.M., Ruonala M.O., Tikkanen R. (2018). Altered expression of ganglioside metabolizing enzymes results in GM3 ganglioside accumulation in cerebellar cells of a mouse model of juvenile neuronal ceroid lipofuscinosis. Int. J. Mol. Sci..

[160] Koike M., Shibata M., Waguri S., Yoshimura K., Tanida I., Kominami E., Gotow T., Peters C., von Figura K., Mizushima N. (2005). Participation of autophagy in storage of lysosomes in neurons from mouse models of neuronal ceroid-lipofuscinoses (Batten disease). Am. J. Pathol..

[161] Rakheja D., Bennett M.J. (2018). Neuronal ceroid-lipofuscinoses. Transl. Sci. Rare Dis..

[162] Harding A.E. (1983). Classification of the hereditary ataxias and paraplegias. Lancet..

[163] Hensiek A., Kirker S., Reid E. (2015). Diagnosis, investigation and management of hereditary spastic paraplegias in the era of next-generation sequencing. J. Neurol..

[164] Allison R., Edgar J.R., Pearson G., Rizo T., Newton T., Günther S., Berner F., Hague J., Connell J.W., Winkler J. (2017). Defects in ER–endosome contacts impact lysosome function in hereditary spastic paraplegia. J. Cell Biol..

[165] Robinson M.S. (2015). Forty years of clathrin-coated vesicles. Traffic (Copenhagen, Denmark).

[166] Hirst J., Borner G.H., Edgar J., Hein M.Y., Mann M., Buchholz F., Antrobus R., Robinson M.S. (2013). Interaction between AP-5 and the hereditary spastic paraplegia proteins SPG11 and SPG15. Mol. Biol. Cell..

[167] Hirst J., Edgar J.R., Esteves T., Darios F., Madeo M., Chang J., Roda R.H., Dürr A., Anheim M., Gellera C. (2015). Loss of AP-5 results in accumulation of aberrant endolysosomes: Defining a new type of lysosomal storage disease. Hum. Mol. Genet..

[168] Khundadze M., Kollmann K., Koch N., Biskup C., Nietzsche S., Zimmer G., Hennings J.C., Huebner A.K., Symmank J., Jahic A. (2013). A hereditary spastic paraplegia mouse model supports a role of ZFYVE26/SPASTIZIN for the endolysosomal system. PLoS Genetics.

[169] Renvoisé B., Chang J., Singh R., Yonekawa S., FitzGibbon E.J., Mankodi A., Vanderver A., Schindler A.B., Toro C., Gahl W.A. (2014). Lysosomal abnormalities in hereditary spastic paraplegia types SPG15 and SPG11. Ann. Clin. Transl. Neurol..

[170] Hirst J., Itzhak D.N., Antrobus R., Borner G.H.H., Robinson M.S. (2018). Role of the AP-5 adaptor protein complex in late endosome-to-Golgi retrieval. PLoS Biology.

[171] Boutry M., Branchu J., Lustremant C., Pujol C., Pernelle J., Matusiak R., Seyer A., Poirel M., Chu-Van E., Pierga A. (2018). Inhibition of lysosome membrane recycling causes accumulation of gangliosides that contribute to neurodegeneration. Cell Rep..

[172] Branchu J., Boutry M., Sourd L., Depp M., Leone C., Corriger A., Vallucci M., Esteves T., Matusiak R., Dumont M. (2017). Loss of spatacsin function alters lysosomal lipid clearance leading to upper and lower motor neuron degeneration. Neurobiol. Dis..

[173] Ariga T., Yanagisawa M., Wakade C., Ando S., Buccafusco J.J., McDonald M.P., Yu R.K. (2010). Ganglioside metabolism in a transgenic mouse model of Alzheimer’s disease: Expression of Chol-1alpha antigens in the brain. ASN Neuro..

[174] Molander-Melin M., Blennow K., Bogdanovic N., Dellheden B., Mansson J.E., Fredman P. (2005). Structural membrane alterations in Alzheimer brains found to be associated with regional disease development; increased density of gangliosides GM1 and GM2 and loss of cholesterol in detergent-resistant membrane domains. J. Neurochem..

[175] Pernber Z., Blennow K., Bogdanovic N., Månsson J.E., Blomqvist M. (2012). Altered distribution of the gangliosides GM1 and GM2 in Alzheimer’s disease. Dement. Geriatr. Cogn. Disord..

[176] Chan R.B., Oliveira T.G., Cortes E.P., Honig L.S., Duff K.E., Small S.A., Wenk M.R., Shui G., Di Paolo G. (2012). Comparative lipidomic analysis of mouse and human brain with Alzheimer disease. J. Biol. Chem..

[177] Yang D.S., Stavrides P., Mohan P.S., Kaushik S., Kumar A., Ohno M., Schmidt S.D., Wesson D., Bandyopadhyay U., Jiang Y. (2011). Reversal of autophagy dysfunction in the TgCRND8 mouse model of Alzheimer’s disease ameliorates amyloid pathologies and memory deficits. Brain.

[178] Yang D.-S., Stavrides P., Saito M., Kumar A., Rodriguez-Navarro J.A., Pawlik M., Huo C., Walkley S.U., Saito M., Cuervo A.M. (2014). Defective macroautophagic turnover of brain lipids in the TgCRND8 Alzheimer mouse model: Prevention by correcting lysosomal proteolytic deficits. Brain.

[179] Zhou S., Davidson C., McGlynn R., Stephney G., Dobrenis K., Vanier M.T., Walkley S.U. (2011). Endosomal/lysosomal processing of gangliosides affects neuronal cholesterol sequestration in Niemann–Pick disease type C. Am. J. Pathol..

[180] Beutler E., Grabowski G.A., Scriver C.R., Beaudet A.L., Sly W.S., Valle D. (2001). Gaucher disease. The Metabolic and Molecular Bases of Inherited Disease.

[181] Gilbert E.F., Dawson G., Zu Rhein G.M., Opitz J.M., Spranger J.W. (1973). I-cell disease, mucolipidosis II. Pathological, histochemical, ultrastructural and biochemical observations in four cases. Z Kinderheilkd.

[182] Nilsson O., Fredman P., Klinghardt G.W., Dreyfus H., Svennerholm L. (1981). Chloroquine-induced accumulation of gangliosides and phospholipids in skeletal muscles. Eur. J. Biochem..

